# Comparative Genomics of Marine Bacteria from a Historically Defined Plastic Biodegradation Consortium with the Capacity to Biodegrade Polyhydroxyalkanoates

**DOI:** 10.3390/microorganisms9010186

**Published:** 2021-01-16

**Authors:** Fons A. de Vogel, Cathleen Schlundt, Robert E. Stote, Jo Ann Ratto, Linda A. Amaral-Zettler

**Affiliations:** 1 Department of Marine Microbiology and Biogeochemistry, NIOZ Royal Netherlands Institute for Sea Research, P.O. Box 59, 1790 AB Den Burg, The Netherlands; fons.de.vogel@nioz.nl; 2 Faculty of Geosciences, Department of Earth Sciences, Utrecht University, P.O. Box 80.115, 3508 TC Utrecht, The Netherlands; 3 Josephine Bay Paul Center for Comparative Molecular Biology and Evolution, Marine Biological Laboratory, Woods Hole, MA 02543, USA; cschlundt@geomar.de; 4 U.S. Army Combat Capabilities Development Command Soldier Center, 10 General Greene Avenue, Natick, MA 01760, USA; robert.e.stote.civ@mail.mil (R.E.S.); joann.rattoross.civ@mail.mil (J.A.R.); 5 Department of Freshwater and Marine Ecology, Institute for Biodiversity and Ecosystem Dynamics, University of Amsterdam, P.O. Box 94240, 1090 GE Amsterdam, The Netherlands

**Keywords:** biodegradation standard test methods, plastic biodegradation, polyhydroxyalkanoate (PHA) cycle, PHA depolymerases, comparative genomics, plastisphere

## Abstract

Biodegradable and compostable plastics are getting more attention as the environmental impacts of fossil-fuel-based plastics are revealed. Microbes can consume these plastics and biodegrade them within weeks to months under the proper conditions. The biobased polyhydroxyalkanoate (PHA) polymer family is an attractive alternative due to its physicochemical properties and biodegradability in soil, aquatic, and composting environments. Standard test methods are available for biodegradation that employ either natural inocula or defined communities, the latter being preferred for standardization and comparability. The original marine biodegradation standard test method ASTM D6691 employed such a defined consortium for testing PHA biodegradation. However, the taxonomic composition and metabolic potential of this consortium have never been confirmed using DNA sequencing technologies. To this end, we revived available members of this consortium and determined their phylogenetic placement, genomic sequence content, and metabolic potential. The revived members belonged to the *Bacillaceae*, *Rhodobacteraceae*, and *Vibrionaceae* families. Using a comparative genomics approach, we found all the necessary enzymes for both PHA production and utilization in most of the members. In a clearing-zone assay, three isolates also showed extracellular depolymerase activity. However, we did not find classical PHA depolymerases, but identified two potentially new extracellular depolymerases that resemble triacylglycerol lipases.

## 1. Introduction

Each year, an estimated 5 to 13 million metric tons of plastic waste flows into the ocean from land, a figure that is expected to only increase in the future [1]. Due to its durability, plastic waste is accumulating and becoming more visible, increasing its ecological and economic impacts [2]. Governments, industry, academia, and consumers are therefore looking for plastic alternatives and ways of reducing plastic use and waste. This has already prompted the International Convention for the Prevention of Pollution from Ships (MARPOL) [3], a plan of action for preventing waste and marine litter by the United Nations in their Sustainable Development Goals [4], and by the European Union (EU) in setting up a strategy on plastic waste reduction [5]. The EU is currently taking a leading role in bans on oxo-(bio)degradable plastics (petroleum-based plastics designed to fragment faster) and single-use plastics for which alternatives exist (i.e., cotton bud sticks, tableware, and expanded polystyrene packaging material). Next to passing legislation to reduce plastic use, the EU is also implementing extended producer responsibility for litter clean-up (i.e., tobacco filters and fishing gear), and improving waste management and recycling [6,7].

Replacing certain conventional plastics with biodegradable alternatives offers an opportunity to reduce plastic waste accumulation, a concept put forth already decades ago [8] and now seeing renewed interest. Although durability is still a desirable property for some plastics, single-use plastics could be effective targets. While all plastics are fragmented due to weathering by ultraviolet radiation and mechanical action [9], microbes can metabolically utilize biodegradable and compostable plastics and fully degrade them within weeks to months instead of multiple years to even centuries [10]. The organic building blocks of the plastic are used to gain energy and to form new cellular biomass under the formation of carbon dioxide and water in aerobic conditions and also to methane in anaerobic conditions [11]. Biodegradable plastics can, however, be made from both renewable or petroleum-based resources and these plastics are not biodegraded in all environments. Specific conditions are often needed for biodegradation, for instance, industrial composting conditions, where pH, moisture, and the microbial community are controlled, and the temperature reaches over 50 °C for prolonged periods of time. In contrast to industrial composting, natural environments vary widely in microbial concentrations and community composition, temperature, oxygen, sunlight, humidity, nutrient limitations, and in the case of aquatic environments, also hydrostatic pressure [12]. Marine environments further subject plastic debris to both horizontal and vertical transport where they encounter changing conditions and not just a static environment as a final destination [13]. Biodegradability is always connected to a specific environment and this makes it extremely challenging to design, control, and ensure significant biodegradation [11,14].

In order to estimate biodegradation under natural conditions, standard test methods have been developed which provide a way to compare the fates of different forms of plastic materials within a reasonable cost and timeframe [15]. These tests are available from ASTM International, CEN (European Committee for Standardization) and ISO (International Organization for Standardization) for biodegradability of plastics in composting environments (e.g., ASTM D6400 and D6868, EN13432, EN 14995, ISO 17088, and ISO 18606), soil (e.g., ASTM D5988, EN 17033, and ISO 17556) and marine environments (e.g., ASTM D6691 and D7991). The standards require an amount of carbon content being converted to CO_2_ within a specific timeframe, as measured by respirometry. This is >90% within 180 days in an industrial composting facility, >90% after 2 years in soil, and at least 60% within 180 days at 30 °C for marine environments. The tests employ either a natural inoculum or a defined microbial community. A natural inoculum, however, might not be representative of environmental biodegradation, especially if there are no specifications for the natural inoculum source [16]. For optimal standardization, comparability, and reproducibility, a defined microbial community is preferred. However, microbial representatives derived from relevant natural environments are then required. It is a challenge to define a representative consortium, since it is still largely unknown if there is a group of core members colonizing plastics in nature. The plastic-associated microbes seem not only to be dependent on the environment but also geographic location, time, and substrate [17]. A relevant defined consortium should probably contain representatives of all domains of life, including eukaryotes like fungi, which are known colonizers of plastics, in addition to bacteria.

A major pathway in (bio)degradation of polymers, before microbial utilization, is hydrolysis, which leads to chain scission and molecular weight decrease [18]. In natural environments, hydrolysis can occur both abiotically and biotically, the latter via secreted enzymes of microorganisms catalyzing this reaction. Hydrolysable polymers with susceptible chemical bonds include polyanhydrides, polyamides, polyethers, polyesters, and polysaccharides [18], with the best-known examples of biodegradable plastics falling into the latter two categories. Examples of these plastics are: PBAT (polybutylene adipate terephthalate), PBS (polybutylene succinate), PCL (polycaprolactone), PHAs (polyhydroxyalkanoates), PLA (polylactic acid) and starch and cellulose based (co-)polymers. Among the best suitable alternatives for conventional plastics are the PHA-derived plastics, since they show biodegradability in both soil and aquatic environments, and in composting conditions, recently reviewed in [19,20,21]. This in contrast to PLA and PBS that seem to biodegrade poorly in seawater [22,23].

PHAs are not only biodegradable, but also biobased polyesters, that have physicochemical properties comparable to conventional plastics [24,25]. With over 150 described monomers, they are classified according to the number of carbons in their monomers, the position of the hydroxyl group, and the presence of functional groups in their side chains (e.g., phenoxy, phenyl and acetoxy groups) [26]. PHAs that consist of monomers of 3–5 carbon atoms are referred to as short-chain length PHAs or PHAscl, while those comprised of 6–15 carbon atoms are called medium chain length PHAs or PHAmcl. Applications of PHAs are found in packaging and agriculture and their biodegradation capability also makes them interesting for medical and therapeutic applications [27,28,29]. Currently, their production cost is relatively high and tuning of the monomeric composition and the molecular weight is often required to get suitable thermal and mechanical properties, limiting a wider application of the polymer [30]. However, the production of this plastic is predicted to reach over 900,000 tons/year in 2020 [31].

Many bacteria accumulate PHAs as storage compounds of both carbon and energy in response to carbon excess and/or nitrogen or phosphate stress [32]. With the first PHAs already being described in 1926 in *Bacillus megaterium* [33], it is now known that both Gram-positive and Gram-negative bacteria have PHA production capabilities, including members of the genera *Aeromonas*, *Alcaligenes*, *Bacillus*, *Cupriavidus*, and *Pseudomonas* [26]. These various microorganisms can be and are currently utilized and optimized to produce PHAs for the biobased plastics industry [34]. PHAs differ in their biophysiological state, based on whether they occur inside or outside the cell. Intracellular PHA is referred to as native PHA (nPHA) and these granules are amorphous, consisting of a polymer core with a surface layer of structural and functional proteins [35]. Extracellular PHA granules, which are released after cell death and cell lysis, consist of a denatured form of PHA (dPHA), which is semi-crystalline in form and lacks this surface layer. These dPHAs can be scavenged by microorganisms from the environment for utilization [36].

**Figure 1:**
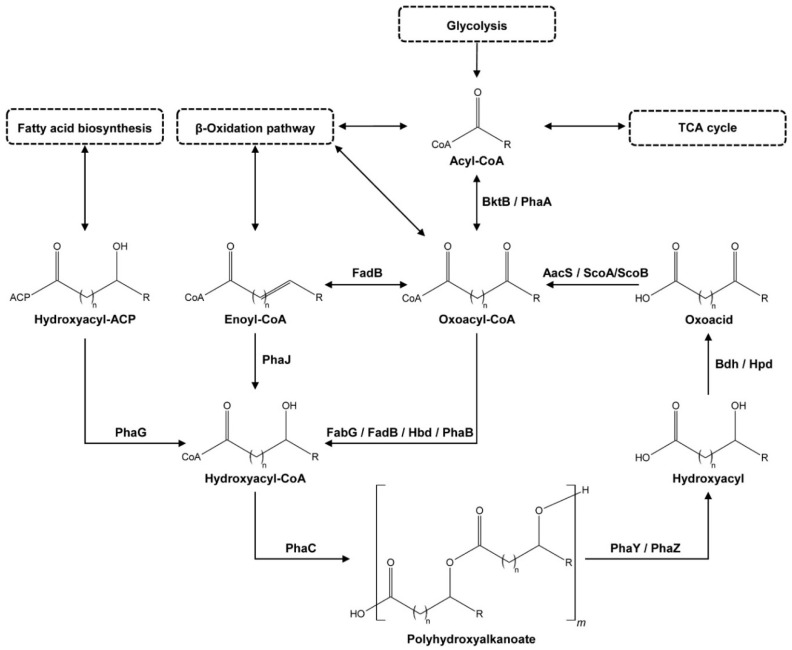
General overview of the polyhydroxyalkanoate (PHA) cycle with examples of enzymes catalyzing the reactions. Enzymes shown are: AacS—acetoacetyl-CoA synthetase, Bdh—3-hydroxybutyrate dehydrogenase, BktB—β-ketothiolase, FabG—3-oxoacyl-[acyl-carrier-protein] reductase, FadB—multifunctional enoyl-CoA hydratase and hydroxyacyl-CoA dehydrogenase, Hbd—3-hydroxybutyryl-CoA dehydrogenase, Hpd—3-hydroxypropionate dehydrogenase, PhaA—acetyl-CoA acetyltransferase, PhaB—acetoacetyl-CoA reductase, PhaC—PHA synthase, PhaG—hydroxyacyl-CoA-[acyl-carrier-protein] transferase, PhaJ—(R)-specific enoyl-CoA hydratase, PhaY—PHA oligomer hydrolase, PhaZ—PHA depolymerase, and ScoA/ScoB—3-oxoacid CoA-transferase subunit A and B, with CoA = coenzyme A and ACP = acyl carrier protein.

There are numerous metabolic pathways known for PHA biosynthesis, most-recently reviewed in Choi et al. [26], which result in various PHA (co-)polymers. A simplified overview of the PHA biosynthetic and degradation pathways, the PHA cycle, is depicted in Figure 1. The cycle starts with an acyl-CoA (coenzyme A) molecule. This acyl-CoA can be generated from unrelated carbon sources via natural biosynthetic pathways. It is, for instance, a product of glycolysis and an intermediate in the TCA (tricarboxylic acid) cycle and the β-oxidation pathway. PhaA (Acetyl-CoA acetyltransferase) and β-ketothiolases like BktB (EC 2.3.1.9/ 2.3.1.16) can convert the acyl-CoA to an oxoacyl-CoA. PhaB (Acetoacetyl-CoA reductase, EC 1.1.1.36) or Hbd (3-hydroxybutyryl-CoA dehydrogenase, EC 1.1.1.157) then hydrolyze the oxoacyl-CoA to a hydroxyacyl-CoA. This step can also be performed by multifunctional enzymes like FabG, a 3-ketoacyl-[acyl-carrier-protein] (ACP) reductase (EC 1.1.1.100) that can also catalyze 3-ketoacyl-CoA to 3-hydroxyacyl-CoA, or FadB, which can act as a hydroxyacyl-CoA dehydrogenase (EC 1.1.1.35) and an enoyl-CoA hydratase (EC 4.2.1.17). The hydroxyacyl-CoA can also be produced from other compounds like enoyl-CoA, also present in the β-oxidation pathway, by PhaJ ((R)-specific enoyl-CoA hydratase, EC 4.2.1.119) or from a hydroxyacyl-[acyl carrier protein] complex, a compound made in fatty acid biosynthesis, by PhaG (hydroxyacyl-CoA-[acyl-carrier-protein] transferase, EC 2.4.1.-). PhaC (PHA synthase, also called PHA polymerase, EC 2.3.1.-), polymerizes the hydroxyacyl-CoA into PHA, the last step in the PHA biosynthesis [26].

In the PHA degradation pathway (see Figure 1), PHA is first depolymerized to monomers by PHA depolymerases (PhaZ, EC 3.1.1.75 and EC 3.1.1.76) and PHA oligomer hydrolases (PhaY, EC 3.1.1.22) to hydroxyacyls [37,38,39]. PhaZs are part of the alpha/beta-hydrolase family, together with enzymes like cutinases, esterases, and lipases [40], and belong to the group of carboxylic ester hydrolases (EC 3.1.1.-) [41]. However, unlike these other enzymes, they have a high diversity in amino acid sequence composition [42]. Both intracellular and extracellular PhaZs exist, with the latter type being required for biodegradation of commercial PHAs and allowing microorganisms to scavenge dPHAs [42]. Similar to PhaZ, PhaY proteins can also be intracellular and/or extracellular, however, they show a higher affinity towards PHA oligomers than polymers [43,44,45,46]. After depolymerization, dehydrogenases like Bdh (3-hydroxybutyrate dehydrogenase, EC 1.1.1.30) [47,48] or Hpd (3-hydroxypropionate dehydrogenase, EC 1.1.1.59) oxidize the hydroxyacyl [49,50]. Coenzyme A synthetases, like AacS (Acetoacetyl-CoA synthetase, EC 6.2.1.16), or 3-oxoacid CoA-transferases like ScoA/ScoB (EC 2.8.3.5) then close the PHA cycle, converting the oxoacid intermediate back to an oxoacyl-CoA molecule [51,52].

In this study, we revisited the historically defined bacterial consortium from the original ASTM marine biodegradation standard test method ASTM D6691: “Standard Test Method for Determining Aerobic Biodegradation of Plastic Materials in the Marine Environment by a Defined Microbial Consortium” [53]. This consortium was developed in the 1990s as a starting point for standardizing inocula used in plastic biodegradation testing. The members of this consortium were selected based on their individual biodegradation capacity of one or multiple biodegradable plastics. This capacity was determined by clearing-zone assays and/or the ability to grow in medium with a specific polymer as the sole carbon source. The tested plastics included different formulations of the PHA co-polymer PHBV (poly(3-hydroxybutyrate-co-3-hydroxyvalerate)), PCL, PVOH (polyvinyl alcohol), cellulose, starch and other polysaccharides [54,55]. This resulted in fourteen suitable isolates for the biodegradation consortium. Marine biodegradation experiments combining eleven members of the consortium, showed positive results for the biodegradation of PHAs, and protein and polysaccharide-based polymers [56,57]. A combination of thirteen of the isolates was used in testing the biodegradation of PHB (poly(hydroxybutyrate)) and PHBV [15,58], and other PHAs of various composition, form, and, crystallinity. In order to identify the members of the consortium, the Biolog Substrate Metabolism System (Biolog, Inc., Hayward, CA, USA), in combination with biochemical methods (i.e., Gram stains) and microscopy, was used originally [54,55]. DNA-sequencing based approaches have hitherto never confirmed the taxonomic composition and metabolic potential of this consortium. This experimentally-confirmed biodegrading consortium offers much-needed insights into the process of biodegradation from a community perspective since our knowledge of plastic biodegradation and the enzymes responsible for doing so is underexplored. We hypothesized that the genomes of several, if not all members, contain genes of the PHA cycle in order to utilize PHA monomers, and that at least one of the consortium members contains genes encoding extracellular hydrolases and/or depolymerases in order to scavenge and break down commercial PHAs. To this end, we revived available members of the original consortium and tested the individual isolates for extracellular depolymerase activity. Furthermore, we determined the phylogenetic placement of the isolates, and performed comparative genomic analysis on six of the consortium members. Four isolate genomes are presented for the first time in this paper. Comparative genomics allowed us to identify PHA cycle genes and assess the biodegradability potential of part of the biodegradation consortium. We identified putative enzyme candidates closely related to extracellular depolymerases and triacylglycerol lipases in the sequenced genomes as likely contributors to PHA degradation.

## 2. Materials and Methods

### 2.1. Microbe Culture Origins and Revival

The original biodegradation consortium, to be employed for ASTM D6691 [53], consisted of up to fourteen members (see Table 1). Eight of the members, NTK009, NTK016B, NTK060, NTK071, NTK072, NTK073, NTK074B, and NTK_Randy, were isolated from experiments in which polymers were exposed to sediment and water collected from Wingaersheek Beach, Gloucester, MA, USA. More specifically, NTK009 was isolated from a PHBV strip exposed to sediment and NTK016B was isolated from sediment during a PCL biodegradation experiment. NTK060, NTK071, NTK072, NTK073 and NTK074B were all isolated from a PCL surface that was exposed to seawater for 8 weeks and NTK_Randy was isolated from a water sample. Five consortium members, isolates NTK029, NTK034, NTK039, NTK045, and NTK049, were isolated from water samples from the Pacific Ocean near Hawaii (USA). Lastly, NTK074Act was isolated from EVOH (ethylene vinyl alcohol) powder evaluated during a respirometry experiment. Unfortunately, seven of the isolates from the consortium were lost since then. The isolates originally identified as *Vibrio proteolyticus* (NTK045) and *Vibrio alginolyticus* (NTK049) were therefore replaced with commercially available strains in 2009. These included *V. proteolyticus* ATCC 15338 = NBRC 13287 [59] and *V. alginolyticus* ATCC 33787 [60] respectively. The other four isolates, NTK009, NTK039, NTK060 and NTK074Act, were not replaced or further retrieved. All the remaining and replaced isolates were provided in 2017 by the U.S. Army Combat Capabilities Development Command Soldier Center (Natick, MA, USA) for analysis undertaken in the present study.

We revived all available isolates from frozen stocks by inoculation on TSY-agar plates (10 g tryptone, 5 g yeast extract, and 15 g agar in 1 L of 75% seawater (salinity ≈ 30, Vineyard Sound, MA, USA) and 25% MilliQ water) and incubation at 30 °C. We then grew liquid cultures from single colonies and both cryopreserved these in 10% DMSO for future work and performed DNA extractions on the harvested cultures.

### 2.2. DNA Extraction and Sanger Sequencing of 16S rRNA Genes

Genomic DNA from each culture was extracted and purified using the Gentra Puregene Yeast/Bacteria Kit (Qiagen, Hilden, Germany), following the manufacturer’s protocols for Gram-positive and/or Gram-negative bacteria when applicable. Genomic DNA was amplified using modified bacterial specific 16S ribosomal RNA (rRNA) gene primers 8F (5′-GTTTGATCCTGGCTCAG-3′) and 1492R (5′-TACCTTGTTACGACTT-3′) [61,62] and directly sequenced at the University of Chicago’s Comprehensive Cancer Center’s DNA Sequencing and Genotyping Facility using their methods. We assembled the resulting forward and reverse sequence reads and manually edited these in Geneious Prime build 29 November 2019 [63]. The resulting consensus sequences were subsequently queried against the nr database at NCBI (National Center for Biotechnology Information), using a BLASTn search [64] to retrieve the sequence and GenBank numbers of the most closely related taxa for phylogenetic placement of NTK sequences.

### 2.3. Phylogenetic Analysis

We searched for additional nearest neighbors of the NTK strains in the SILVA SSU database release 138 Ref NR 99 [65] and aligned them using SINA aligner v1.2.11 [66]. The NTK strain sequences and sequences from the NCBI top BLASTn results not already in the SILVA reference database, were aligned to the database using the command-line version of SINA 1.6.0. We then imported the aligned, arb-formatted sequences into the 138 Ref NR 99 SSU database using ARB v6.0.6 [67], where we selected additional 16S rRNA gene sequences to complete the backbone of the tree. We then exported the resulting alignment and adjusted it manually in Geneious Prime. The shortest sequences were removed, resulting in 70 taxa for phylogenetic reconstruction. The remaining sequences were trimmed and sites containing any gaps were stripped, leaving 1279 phylogenetically informative positions. We then used IQ-TREE (multicore version 1.6.7 for Linux 64-bit, built 23 August 2018) [68] with the ModelFinder flag [69], which selected the K2P + I + G4 model as the best-fit evolutionary model. We chose *Bacillus subtilis* DSM10 as an outgroup and ascertained the confidence of the branching in the tree topology via 1000 bootstrap iterations. The resulting tree was visualized using the Interactive Tree Of Life tool (iTOL, v5.5.1) [70] and further refined in Adobe Illustrator 23.1 (Adobe Systems Inc., San Jose, CA, USA).

### 2.4. Isolate Growth on PHAs and Screening for Extracellular PHA Depolymerase Activity

Growth of individual isolates on PHBV was checked after isolation in the 1990s [54,55]. Next to that, a screening was performed with a non-specific compositional form of PHAs (i.e., a PHA polymer consisting of multiple different hydroxyalkanoates) [unpublished study]. For this screening, liquid cultures were grown in the presence of 0.2 *w*/*v*% PHA film (Imperial Chemical Industries) or 0.2 *w*/*v*% of grounded polymer powder, which was added to a carbon-free mineral salts medium. The medium consisted of 1 g NH_4_Cl, 0.8 g MgSO_4_•7H_2_O, 0.45 g K_2_SO_4_, 12 mL of 1.1 M phosphoric acid, 0.015 g Fe_2_(SO_4_)_3_•7H_2_O and 24 mL trace element solution added to 964 mL distilled water. The trace elements solution consisted of 0.02 g CuSO_4_•5H_2_O, 0.1 g ZnSO_4_•6H_2_O, 0.1 g MnSO_4_•4H_2_O and 2.6 g CaCl_2_•2H_2_O in 1 L distilled water. Two of the isolates, NTK009, and NTK016B, would not grow on the above media and were rescreened along with others using a marine-specific defined media consisting of 2 g NH_4_Cl, 2 g MgSO_4_•7H_2_O, 0.05 g K_2_SO_4_, 0.5 g KNO_3_, 500 mL synthetic seawater and 500 mL distilled water. Presence of growth was determined visually (powder cultures) and by measuring weight loss of the films.

Here, we tested extracellular PHA depolymerase activity for the individual isolates on 25 mm Petri plates with f/2-silicate medium [71], made with low-nutrient seawater with 10 g/L agar added. A thin PHA layer was added on top of the agar, by pouring 10 mg of PHA (Goodfellow—PH326300—3 mm granules—Extrusion Grade) dissolved in 3 mL chloroform. The chloroform was then evaporated, while plates were rocking at 10 rev/min on a rocking shaker, leaving a solid PHA layer. We placed the cultures in Marine Broth 2216 (BD—Difco) from cryopreserved stocks, and grew them overnight, shaking at 200 rpm in a shaking incubator at 30 °C. From the revived cultures, 20 µL of the liquid cultures were spread onto the PHA-plates and left to air dry, before being incubated at 30 °C. After growth was confirmed, plates were stored at 4 °C and checked at regular intervals for clearing zones.

### 2.5. Whole Genome Sequencing, Assembly, and Annotation

We selected four NTK strains for whole-genome sequencing: NTK016B, NTK071, NTK072, and NTK074B. The genomic DNA (68 ng from NTK074B and 100 ng from the remaining cultures) was sheared to 275 bp on a Covaris S220 focused-ultrasonicator (Covaris, Inc., Woburn, MA, USA). We purified sheared samples using Agencourt AMPure XP magnetic beads (Beckman Coulter, Inc., Danvers, MA, USA). The genomic libraries were constructed using the Ovation Ultralow DR Multiplex System V2 1–8 library construction kit (NuGEN Technologies Inc., San Carlos, CA, USA). The libraries were pooled equimolarly and size-selected using a Pippin Prep (Sage Science, Inc., Beverly, MA, USA), targeting a size of 390 bp. We purified the size-selected pool again using AMPure magnetic beads in a 1:1 sample to bead ratio. Size, quantitation, and quality were confirmed using a 2100 Bioanalyzer DNA High Sensitivity chip (Agilent Technologies, Santa Clara, CA, USA). The genomic library pool was further verified through qPCR using KAPA SYBR-FAST for Illumina platforms (Kapa Biosystems, Inc., Wilmington, MA, USA). An Illumina NextSeq V2 Mid Output Sequencing kit (300 cycles) was used, and based on the qPCR results, the genomic library pool was diluted to 2 pM, denatured, and clustered according to the Illumina NextSeq protocol. PhiX DNA was added at 1% for quality control purposes. The samples were then run on an Illumina NextSeq 500 instrument at the Marine Biological Laboratory’s W.M. Keck Sequencing Facility.

We checked the quality of the obtained raw reads using FastQC v0.11.3 (Babraham Bioinformatics, Babraham Institute, Cambridge, United Kingdom). Reads were paired after low-quality bases were trimmed using Trimmomatic v0.35 [72], with settings HEADCROP:6 LEADING:28 TRAILING:28 SLIDINGWINDOW:4:20 MINLEN:40. Genomes were then assembled from the FastQ files, using SPAdes v3.11.1 [73] with the “careful” flag included. Both paired reads and unpaired reads were used as input and maximum k-mer length iterations were chosen resulting in an average coverage above 50. For NTK016B these parameters were: 21, 33, and 55; for NTK074B these were: 21, 33, 55, and 77; and for NTK034 and NTK071 these were: 21, 33, 55, 77, and 99.

We retrieved genome sequences for *V. proteolyticus* ATCC 15338 = NBRC 13287 (Accession no. BATJ01000000: BATJ01000001-BATJ01000050) and *V. alginolyticus* ATCC 33787 (Accession no. chromosome 1: CP013484, chromosome 2: CP013485, plasmid pMBL96: CP013488, plasmid pMBL128: CP013486 and plasmid pMBL287: CP013487) from NCBI. All genomes were annotated using the RASTtk pipeline, which makes use of the FIGfams database [74,75,76].

### 2.6. Pangenomic Analysis

We performed comparative genomics analysis using the pangenomics workflow in Anvi’o v6.1, “esther” [77,78]. We chose close relatives of the NTK strains based on our 16S rRNA gene phylogenetic analyses, genome completion level, type strain, and environmental source (e.g., marine) where feasible. As with the NTK strains, gene functions were annotated using RASTtk and imported into Anvi’o. Searching for amino acid sequence similarity was performed by Anvi’o using NCBI BLASTp [64]. Genome completeness was calculated with the anvi-run-hmms module of Anvi’o, which is based on curated single-copy core genes. Figures were made with the Anvi’o interactive display module and further adjusted in Adobe Illustrator 23.1.

### 2.7. Analysis of Metabolic Potential

We used Anvi’o to search our annotated genomes for the presence of genes encoding key enzymes in the PHA biosynthetic and degradation pathways to predict the PHA metabolic potential of the NTK community members. We also used SignalP-4.1 [79] for Gram-negative or Gram-positive bacteria, when applicable, to search for signal peptides in PHA hydrolases and depolymerases we found to ascertain whether the proteins were secreted. We further refined our search strategy for the two strains that showed clearing zones on the PHA plates, *V. proteolyticus* ATCC 15338 = NBRC 13287 and *Bacillus* sp. NTK074B.

For the refined search, we performed a functional analysis of protein domains using InterProScan 5.20–59.0 [80], with InterPro member databases CATH-Gene3D 3.5.0 [81], Pfam-30.0 [82], PRINTS-42.0 [83] and SUPERFAMILY-1.75 [84]. Together with InterProScan, we ran SignalP-4.1 again, to confirm secretion. A shortlist of extracellular depolymerase candidates was made based on the proteins having both a predicted signal peptide and alpha/beta hydrolase fold domain, indicative of the protein belonging to the alpha/beta hydrolase superfamily, to which the PHA depolymerases belong. We then performed a motif-based search in Geneious Prime for the conserved features of extracellular PHA depolymerases. These are the pentapeptide lipase box (amino acid sequence: GXSXG, AHSMGV, A[I,T]S[S,T]G or AHSXG), the oxyanion pocket (PXXXXHG or HGC), and the amino acids of the catalytic triad, serine (part of the lipase box), aspartic acid and histidine [38]. We then checked for the presence of these candidates in other NTK strains against the calculated gene clusters from Anvi’o.

We constructed alignments of the conserved features in the protein candidates with related extracellular PHA depolymerases from the PHA Depolymerase Engineering Database [42], including an experimentally validated PhaZ [85]. Closely related secreted lipases as determined by Oh et al. [41] and recently described extracellular lipases from *Acinetobacter* spp., that can potentially depolymerize both scl- and mcl-PHAs [86], were also included in the alignment. These alignments were made manually in Geneious Prime and further refined in Adobe Illustrator 23.1.

## 3. Results

### 3.1. Isolate Identification

The original ASTM D6691 biodegradation consortium was identified using the Biolog Substrate Metabolism System (Biolog, Inc., Hayward, CA, USA), along with biochemical methods (i.e., Gram stains) and microscopy [54,55]. This resulted in the identification of the isolates to the genus and sometimes to the species level, see Table 1. The identities of the strains were, however, never confirmed with marker gene sequencing. We revived nine isolates from the consortium to perform this. The original identities of the revived strains included: *Pseudoalteromonas haloplanktis* (NTK029), *V. furnissii* (NTK072), and two *Bacillus* species (NTK071 and NTK_Randy), with one identified as a *B. megaterium*. For three other revived strains (NTK016B, NTK034, and NTK074B), the original identities were unknown. Two replacement strains were also revived. These strains replaced the original isolates NTK045 and NTK049, already at the U.S. Army Combat Capabilities Development Command Soldier Center in Natick. These were the isolates *V. proteolyticus* ATCC 15338 = NBRC 13287 and *V. alginolyticus* ATCC 33787 respectively.

We determined the identity of the revived isolates by sequencing of the nearly complete 16S rRNA gene (see Table 1). Isolates were assigned to three bacterial families: *Bacillaceae*, *Rhodobacteraceae*, and *Vibrionaceae*. After a BLASTn search, isolate NTK016B showed 97% identity (99.72% coverage) with the roseobacter bacterium *Rhodobacter* sp. R18. This closely related *Rhodobacter* strain was isolated from a *Nannochloropsis oculata* algal culture and can inhibit the growth of *Vibrio anguillarum*, a fish pathogen [87]. NTK029 and NTK_Randy returned hits with equal coverage and identity scores to various members from the *Bacillus cereus* group, including *B. anthracis*, *B. albus*, *B. cereus*, *B. nitratireducens*, *B. paranthracis*, *B. thuringiensis*, *B. tropicus*, and *B. wiedmannii*. While the taxonomic assignment of *Bacillus* members in this group based on the 16S rRNA marker gene is challenging, the identity of NTK029 did not correspond to the original one. Originally it was identified as *Pseudoalteromonas haloplanktis*, a Gram-negative proteobacterium and not a Gram-positive firmicute. NTK034 shared the highest sequence similarity to *Bacillus oceanisediminis* 2691, a strain isolated from an intertidal marine sediment on the Yellow Sea coast of South Korea that contains a high amount of heavy metal resistance genes [88]. The obtained 16S rRNA gene sequences of the *V. proteolyticus* and *V. alginolyticus* strains were not 100% identical to the purchased ATCC strains because they contained ambiguous nucleotides. This is likely the result of microheterogeneities in their gene copies. Vibrios have 10 copies of the 16S rRNA gene on average, according to the ribosomal RNA operon database [89]. The ambiguous nucleotides of our sequences corresponded to the heterogeneity of the 16S rRNA gene sequences deposited in NCBI. The 16S rRNA gene sequence of NTK071 had the highest similarity to *Bacillus* sp. N1-1, which is capable of degrading κ-selenocarrageenan, a selenium polysaccharide, and was isolated from a deep sea cold seep marine sediment in the South China Sea [90]. NTK072 had equal top hits to tens of strains of *Bacillus atrophaeus*, all with a 100% coverage and identity, but not with *V. furnissii*, as originally assigned based on non-molecular approaches. The top BLASTn hit of NTK074B was a cadmium tolerant *Bacillus vietnamensis* 151-6, isolated from cadmium-contaminated soil from a former industrial site in China [91]. The 16S rRNA gene sequences are deposited under GenBank accession numbers MW435594-MW435596 and in CPXXXXXXX-CPXXXXX. Associated metadata are specified in Supplementary Tables S9 and S10.

### 3.2. Phylogenetic Placement of NTK Sequences

**Figure 2:**
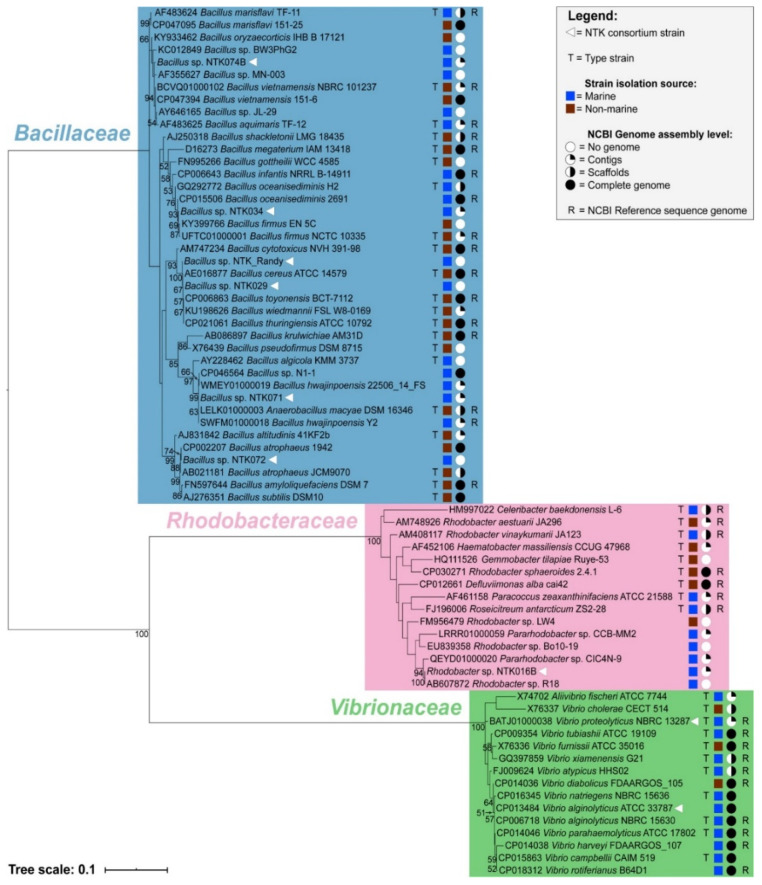
Maximum Likelihood inferred phylogenetic analysis of the NTK consortium isolates and close relatives, based on the 16S rRNA marker gene sequence. Bootstrap support values above 50% are shown on the nodes and GenBank accession numbers are noted next to the strains. Information on the right provides the following: type strain information, isolation source (marine versus non-marine), genome assembly level available, and representative genome status for a given taxon. The evolutionary distance is indicated by the scale bar.

The phylogenetic placement of the NTK consortium isolates can be seen in Figure 2. Additional metadata associated with the microorganisms are also shown: type strain designation, isolation source, genome availability in NCBI with its assembly level, and an indication of whether it is a representative genome for the species. These data are further specified in Supplementary Tables S1–S3, S9 and S10.

The NTK isolates fell into three bacterial families: *Bacillaceae*, *Rhodobacteraceae*, and *Vibrionaceae*. The *Bacillaceae* isolates branched among five different *Bacillus* groups in our phylogeny: NTK_Randy and NTK029 branched among the *B. cereus* group, while NTK034 shared most recent common ancestry with *B. oceanisediminis* strains and members of the *B. firmus* group. *Bacillus* sp. NTK071 was most closely related to *B. hwajinpoensis* strains and *Anaerobacillus macyae*, while NTK072 showed most recent common ancestry with *B. atrophaeus* strains (bootstrap support 74 and 88). The branching of NTK074B was not well-resolved between *B. marisflavi*, *B. oryzaecorticis* and *B. vietnamensis* strains. *Rhodobacter* sp. NTK016B displayed high branch support for placement with the aforementioned *Rhodobacter* sp. R18 strain and formed part of a larger well-supported cluster with *Pararhodobacter* sp. strain CIC4N-9 (bootstrap support 94), a bacterium isolated from deep-sea water in the Indian Ocean [92]. *V. alginolyticus* ATCC 33787 branched with the type strain of *V. alginolyticus*, NBRC 15630, but the position of *V. proteolyticus* NBRC 13287 was poorly resolved among other vibrios owing a lack of resolving power among 16S rRNA gene sequences to differentiate between *Vibrio* species in general.

### 3.3. Isolate Growth on PHAs and Screening for Extracellular Depolymerase Activity

The isolates NTK074B and NTK_Randy showed positive growth when grown in liquid culture with PHAs as the only carbon source, see Table 2, indicating a full metabolic potential to utilize PHAs. After we incubated the NTK isolates on PHA-covered agar culture plates for 4 days at 30 °C, colonies started to become visible for all strains except NTK029, which was not tested. The number of colonies ranged from two to more than 100 colonies. However, no clearing zones were observed in the 4-day timeframe, which would indicate high activity of extracellular depolymerases. The plates were therefore stored at 4 °C and checked again after 2 months. Clearing zones of several millimeters around the colonies were then observed for *V. proteolyticus* ATCC 15338 = NBRC 13287 and for NTK072 and NTK074B, two *Bacillus* strains, see Table 2.

### 3.4. Whole-Genome Sequencing

While the two *Vibrio* strains were already sequenced, four NTK strains were selected for whole-genome sequencing: *Rhodobacter* sp. NTK016B and *Bacillus* spp. NTK034, NTK071 and NTK074B (Table 2). Table 3 summarizes the genome features after assembly and annotation in RASTtk. Genome sizes varied from 4,164,462 bp (NTK071) to 5,599,963 bp (NTK034), with a GC-content of around 40% for the *Bacillus* genomes and of about 65% for the *Rhodobacter* genome. The number of contigs varied between 100 (NTK034) to 446 (NTK074B) in total, with half of the genome length covered by four to eight contigs (L50 value). The N50 value was between 225,829 for the smallest genome, to 423,522 for the largest genome and the average coverage of all genomes was above 50 reads. Using the RASTtk pipeline, 4385 to 6087 genes and 46–128 RNAs were annotated per genome. The RASTtk annotated genomes are available online *via* https://rast.nmpdr.org/ by logging in as guest and can be found under ID numbers 6666666.521526 (*Rhodobacter* sp. NTK016B), 6666666.521528 (*Bacillus* sp. NTK034), 6666666.521530 (*Bacillus* sp. NTK071) and 6666666.521531 (*Bacillus* sp. NTK074B). The raw sequence reads are deposited in the NCBI Sequence Read Archive under BioProject PRJNA649735 with sample accession numbers SRR12354198 to SRR12354201. Annotated genomes are also deposited in INSDC under references CPXXXXXX-CPXXXXXX, and associated metadata are specified in Supplementary Table S10.

### 3.5. Pangenomic Analysis

**Figure 3:**
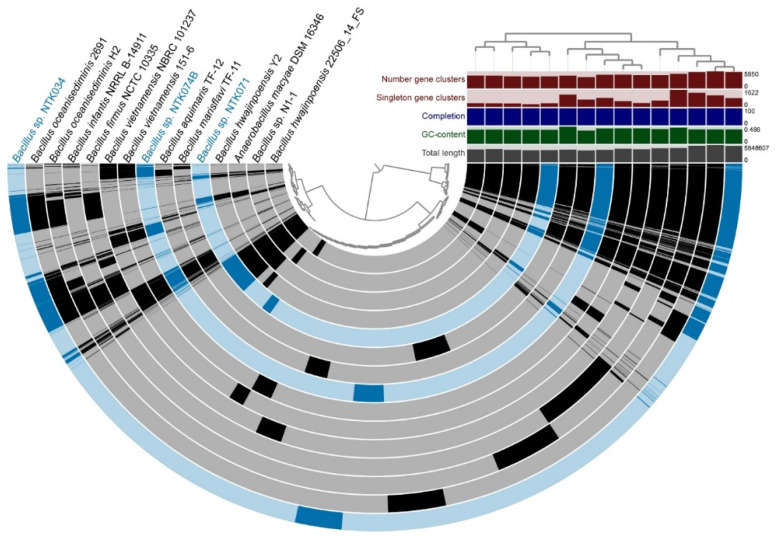
Genome comparison of the three sequenced NTK *Bacillus* spp. genomes (NTK034, NTK071 and NTK074B) in blue, with closely related neighbors in grey. The semicircles show gene presence (dark color) and absence (light color). Alignment of the genomes is based on gene clustering, with the genome order based on the gene cluster presence/absence tree, shown in the upper right corner. The dendrogram in the center represents the hierarchy in gene clustering using Euclidean distance and Ward linkage. Genome properties shown: “Number gene clusters” represents the total number of gene clusters found in the genome; “Singleton gene clusters” represents the number of genes found in only one genome, “Completion” in (%) is calculated based on single-copy genes, “GC-content” shows the average guanine and cytosine nucleotide content and “Total length” is the genome length in base pairs.

**Figure 4:**
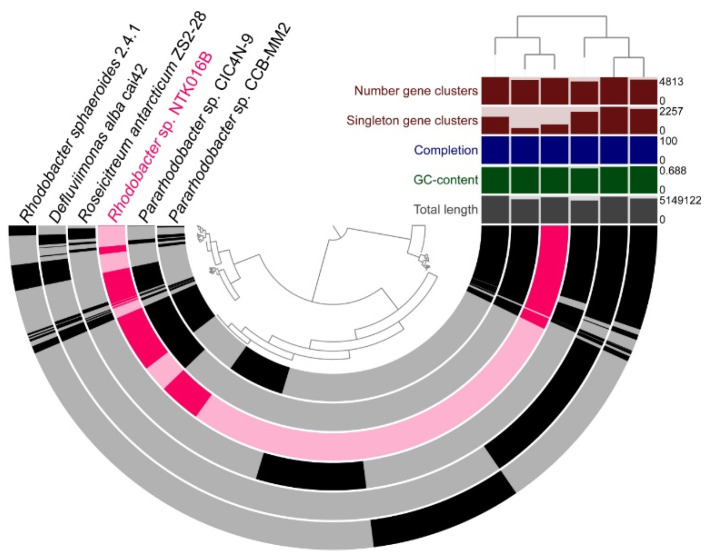
Genome comparison of isolate *Rhodobacter* sp. NTK016B, highlighted in pink, with *Rhodobacteraceae* neighbors with sequenced genomes in grey. The semicircles show gene presence (dark color) and absence (light color). Gene clustering and genome alignment, order, and properties are presented as in Figure 3.

**Figure 5:**
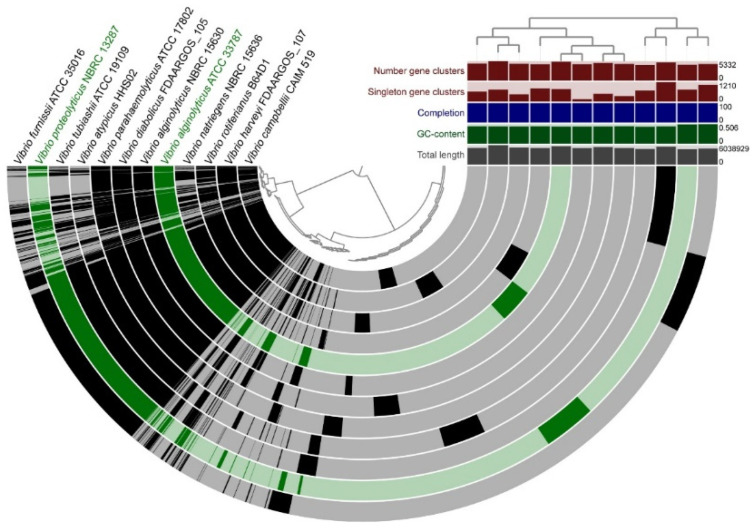
Genome comparison of *V. proteolyticus* NBRC 13287 and *V. alginolyticus* ATCC 33787 (in green), with closely related neighbors (in grey). The semicircles show gene presence (dark color) and absence (light color). Gene clustering and genome alignment, order, and properties are presented as in Figure 3.

The genome relatedness of the sequenced NTK isolates with close neighbors was examined by a pangenomic analysis. Figure 3, Figure 4 and Figure 5 show the genomic alignment of the genomes, based on gene clustering, with the genome order based on gene cluster presence/absence. Genome accession information can be found in Supplementary Table S3. The estimated completeness of the genomes from all the NTK consortium members was 100%, as predicted from the presence of single-copy genes by Anvi’o. Exact numbers for the genome properties can be found in Supplementary Tables S4–S6. The whole-genome comparisons of the three sequenced *Bacillus* spp. genomes (NTK034, NTK071 and NTK074B), with 12 nearest neighbors are shown in Figure 3. As in our 16S rRNA phylogenetic reconstruction, the isolates and nearest neighbors clustered into three groups, based on the gene cluster presence/absence tree.

Figure 4 shows the whole-genome comparison of NTK016B (*Rhodobacter* sp.) with five closely related neighbors that were chosen based on phylogenetic analysis of the 16S rRNA gene. As with the phylogenetic placement, comparative genomics confirmed the close relationship of NTK016B with the *Pararhodobacter* sp. strains CICN4N-9 and CCB-MM2.

The genome sequences of the two NTK consortium vibrios, *V. proteolyticus* NBRC 13287 and *V. alginolyticus* ATCC 33787, were already available in public databases. Figure 5 shows the whole-genome comparison of the *Vibrio* strains with 10 close neighbors with sequenced genomes. The Anvi’o display shows a large portion of shared gene clusters among all the chosen *Vibrio* species. The gene cluster presence/absence tree topology is similar to that of the 16S rRNA gene tree.

### 3.6. Analysis of Metabolic Potential

The PHA metabolic potential of the NTK consortium members was assessed by searching for the presence of genes encoding key enzymes in the PHA biosynthetic and degradation pathways, which are depicted in Figure 1. The results of this search, together with the existence of these genes in closely related species, are shown in Table 4. We found genes encoding for a full PHA biosynthetic capacity in all NTK strain genomes, going from an acyl-CoA to PHA, via an oxoacyl-CoA (by PhaA or BktB) and a hydroxyacyl-CoA (by FabG, FadB, Hbd or PhaB). The only exception to this was strain NTK071, which seemed to be missing the PHA synthase gene (*phaC*). This gene was also missing in the close relatives *Anaerobacillus macyae*, the *B. hwajinpoensis* strains, and *Bacillus* sp. N-1-, the latter with a closed genome. The genes *phaJ* (Hydroxyacyl-CoA-acyl carrier protein transferase) and *phaG* ((R)-specific enoyl-CoA hydratase), which provide an alternative for synthesizing the PHA monomer, were not detected in any of the genomes.

PHA degradation is initiated by PhaZ and PhaY, PHA depolymerase and PHA oligomer hydrolase, respectively. While the PhaZ and PhaY encoding genes were found in some genomes, they were missing in all the consortium members, except for *Rhodobacter* sp. NTK016, which had an annotated *phaZ* gene (see Table 4). Most of the annotated *phaZ* genes were predicted to encode an intracellular PhaZ, based on the absence of a signal peptide in the amino acid sequence. The exceptions to this were PhaZ from *Bacillus infantis* NRRL B-14911 and *Anaerobacillus macyae* DSM 16346. An extracellular depolymerase of *Rhodobacter sphaeroides* 2.4.1, deposited in the PHA Depolymerase Engineering Database [42], was also annotated, however, SignalP did not identify a signal peptide in the amino acid sequence. The *phaY* gene was only annotated in one genome, *Vibrio natriegens* NBRC 15636, and was predicted to be extracellular.

Going from the depolymerized PHA, the monomer (a hydroxyacyl) is converted back to an oxoacyl-CoA, via an oxo-acid, to close the PHA cycle. This is catalyzed sequentially by Bdh or Hpd and AacS or ScoA/ScoB. All genomes had *bdh* and either *aacS* or *scoA* and *scoB* annotated. The *Pararhodobacter* sp. strains and NTK016B had both the *aacS* and *sco* genes. The exceptions were the vibrios, which were all missing the *bdh* gene. After further inspection of these genomes, we found that they did contain *hibdh*, encoding 3-hydroxyisobutyrate dehydrogenase (EC 1.1.1.31). The *hpd* gene was not found in any of the annotated genomes. *Bacillus infantis* NRRL B-14911, *B. vietnamensis* NBRC 101237, *B. vietnamensis* 151-6, *Rhodobacter* sp. NTK016B and the other *Rhodobacteraceae* species all possessed the necessary metabolic repertoire to both synthesize and degrade PHAs intracellularly based on our functional annotation.

Using the refined search for extracellular depolymerases in the strains that showed clearing zones on the PHA plates, *Bacillus* sp. NTK074B and *V. proteolyticus* NBRC 13287, we found two PhaZ candidates. These candidates contained a signal peptide, alpha/beta hydrolase fold domain, a conserved pentapeptide lipase box, oxyanion pocket, and the amino acids of the catalytic triad. These are all the criteria that known PHA depolymerases (PhaZs) contain. In *Bacillus* sp. NTK074B this candidate gene was annotated as a hypothetical protein and in *V. proteolyticus* NBRC 13287 as a lipase precursor (EC 3.1.1.3). Based on the gene clustering from Anvi’o, the former protein was conserved in close relatives *B. aquimaris* TF-12, *B. marisflavi* TF-11, *B. vietnamensis* 151-6 and *B. vietnamensis* NBRC 101237. The latter protein was conserved in all *Vibrio* genomes from the pangenomic analysis, except for *V. natriegens* NBRC 15636 and *V. rotiferianus* B64D1. Thus, it was also present in *V. alginolyticus* ATCC 33787 (85.2% amino acid sequence similarity) even though this strain did not show clearing zones. The amino acid sequences of the extracellular PhaZ candidates are given in Supplementary Table S7.

**Figure 6:**
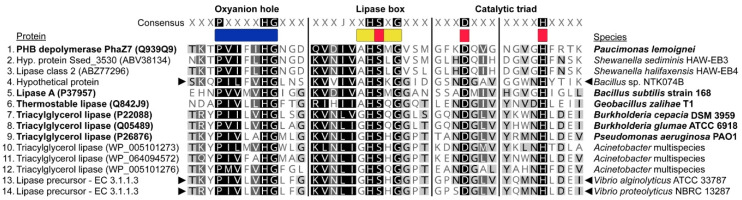
Alignment of the conserved features in known extracellular PHA depolymerases and closely related lipases, together with those from the extracellular depolymerase candidates of the NTK biodegradation consortium members (marked by ►◄). Shown are the oxyanion hole (blue), lipase box (yellow), and amino acid residues of the catalytic triad (red). Proteins from species with experimentally validated structural features are marked bold. Highlighted amino acids in the alignment at specific positions have similar physicochemical properties.

We aligned the conserved features of known extracellular PHA depolymerases and closely related lipases, with our extracellular PhaZ candidates. The order of the conserved features in all these sequences was: (1) oxyanion hole; (2) lipase box with the serine (S) of the catalytic triad; and, (3) the remaining amino acids of the catalytic triad, aspartic acid (D) and histidine (H). Furthermore, all these proteins had either a predicted signal peptide (SignalP 4.1) or were experimentally proven to be secreted. The alignment is shown in Figure 6 and available protein structure information can be found in Supplementary Table S8. Of the known extracellular PhaZ proteins, our candidates were most similar to the experimentally validated PhaZ7 from *Paucimonas lemoignei* [85]. For this protein, the structure has also been determined, confirming the positions of the conserved features [93,94,95]. Two other proteins were categorized with PhaZ7 from *P. lemoignei* in the PHA Depolymerase Engineering Database [42]. These were from *Shewanella sediminis* and *Shewanella halifaxensis*, however, these have no experimental data associated with them. Secreted triacylglycerol lipases were added to the alignment based on their close evolutionary relationship with PhaZ7, as determined by Oh et al. [41]. These lipases were: lipase A from *Bacillus subtilis*, a thermostable lipase from *Geobacillus zalihae* and triacylglycerol lipases from *Burkholderia cepacia*, *Burkholderia glumae*, and *Pseudomonas aeruginosa*. These also all have had their protein structures determined, confirming the positions of the conserved features. The triacylglycerol lipases from *Acinetobacter* spp. were described earlier as possible PHA depolymerases by Sharma et al. [86] and also these proteins had the conserved features in their amino acid sequence. The oxyanion hole in all the proteins had the PXXXXHG amino acid motif. The lipase box found in the hypothetical protein of *Bacillus* sp. NTK074B was most similar to the PhaZs deposited in the PHA Depolymerase Engineering Database, lipase A, and the thermostable lipase, having the amino acid motif AHSXG. The lipase precursors from our *Vibrio* spp. on the other hand, had a lipase box more similar to the triacylglycerol lipases, with a GHSXH amino acid motif.

## 4. Discussion

As a starting point for standardizing inocula used in plastic biodegradation testing, a marine biodegradation consortium was developed in the 1990s for the original ASTM D6691 plastic biodegradation test [53]. The members, all bacteria, were originally identified using the Biolog Substrate Metabolism System, in combination with biochemical methods (i.e., Gram stains) and microscopy [54,55]. In this study, we used high-throughput sequencing to determine that the revived consortium members consisted of one *Rhodobacter* sp. (NTK016B), and 6 *Bacillus* spp. (NTK029, NTK034, NTK071, NTK072, NTK074B and NTK_Randy), in addition to the lost isolate replacement bacteria *Vibrio proteolyticus*, and *Vibrio alginolyticus*. In doing so we found that the original identification of strains was not always correct: NTK029 is not a *Pseudoalteromonas haloplanktis*, NTK072 is not a *Vibrio furnissii*, and NTK071 is closely related to *B. hwajinpoensis* strains and not to *B. megaterium*. The Biolog Substrate Metabolism System relies on oxidation of carbon sources and has been reported to lead to incorrect identification of strains, especially on the species level [96]. However, the 16S rRNA gene was not always sufficient for determining the identity of our isolates down to the species level either. This was the case for NTK029, NTK072, and NTK_Randy. The phylogenetic position of NTK072 was with *Bacillus atrophaeus*, while NTK029 and NTK_Randy were both placed within the *B. cereus* group. Although the latter group includes several well-described terrestrial species, it also contains species from marine environments [97]. Phylogenetic inference showed that NTK034 shared the most recent common ancestry with members of the *B. firmus* group and NTK074B was branching alongside *B. marisflavi* and *B. vietnamensis* strains. *Rhodobacter* sp. NTK016B displayed high branch support for placement with a *Rhodobacter* sp. strain and formed part of a larger, well-supported cluster with *Pararhodobacter* sp. strains. *Vibrio alginolyticus* ATCC 33787 branched among the type strain of *V. alginolyticus* NBRC 15630, but the position of *V. proteolyticus* NBRC 13287 was poorly resolved among other vibrios owing a lack of resolving power among 16S rRNA gene sequence between *Vibrio* species in general.

The nine consortium isolates that we revived all belong to the *Bacillaceae*, *Rhodobacteraceae*, and *Vibrionaceae* bacterial families. The complete original consortium was purported to also include *Actinomycete* sp., *Pseudomonas* sp., *Vibrio campbellii*, *Xanthomonas campestris*, and *Zoogloea* sp. (see Table 1 and [15]). While the current consortium might seem limited in diversity, the identified families are often mentioned in the literature related to colonization and (bio)degradation of plastics. Some *Bacillus* spp. show the degradation of conventional plastics in laboratory tests [98], but there is no mention of this genus being enriched on plastic surfaces in published environmental surveys. *Rhodobacteraceae*, on the other hand, are known surface colonizers of plastics and are often found in coastal areas, during early colonization [99,100,101,102,103,104]. Vibrios multiply rapidly under favorable conditions and can dominate conventional plastic surfaces [102,103,105,106]. A recent meta-analysis of metagenomes by Viljakainen and Hug [107] analyzed the distribution of PHA depolymerases in microbial communities from diverse aquatic, terrestrial, and waste management systems. Putative PHA depolymerases were predicted in Firmicutes, which includes the genus *Bacillus*, Gammaproteobacteria, that includes the genus *Vibrio*, and in the order Rhodobacterales, among others. The results of Viljakainen and Hug indicated that extracellular PHA depolymerases are globally widespread, but unevenly distributed, with the majority of aquatic environments not having any PHA depolymerases. However, even though the depolymerization genes were not always detected in some aquatic environments, the presence of plastic waste can select for low-abundance microbial community members with degradation capacity [17]. These genes would then not be detected in metagenomes that did not target these substrates, as was the case in this study.

The original ASTM D6691 consortium has been used among others in testing the biodegradation of PHB and PHBV [15,58], and other PHAs of various composition, form, and crystallinity. One important aspect of our study shows that individual members of the original consortium may not have been capable of biodegradation in the absence of other members. Individual members of the consortium were tested for a biodegradation capability by growing them in a medium with PHAs as the only carbon source. Only NTK074B and NTK_Randy demonstrated this biodegradation capability on their own. After incubating the NTK isolates on PHA-covered culture plates, growth was observed for all strains, except NTK029, which was not tested. Clearing zones, indicating extracellular depolymerase activity, were observed for *V. proteolyticus* NBRC 13287, and *Bacillus* spp. NTK072 and NTK074B. With the exception of NTK074B, which showed a positive result in both tests, it could be that select members of the consortium can depolymerase the extracellular polymer (i.e., *V. proteolyticus* NBRC 13287 and NTK072), while other members can utilize the hydroxyalkanoate monomers intracellularly. The discrepancy between these two growth experiments, as was the case for NTK_Randy, could be explained by a difference in the form of PHAs used. Chemical composition [108,109,110,111] and structure (i.e., crystallinity) [112,113,114,115] have been shown to influence the biodegradability of PHA polymers. Further processing of PHA stock material, like casting the PHA films for the clearing zone plate assay, also influences crystallinity [116,117] and thus biodegradability.

To further investigate the metabolic potential, we performed whole-genome sequencing on some of the members, combined with a pangenomic analysis. We hypothesized that the genomes of at least one of the consortium members contained genes encoding extracellular hydrolases and/or depolymerases in order to scavenge and break down commercial PHAs, and that several, if not all members, possessed genes of the PHA cycle in order to utilize PHA monomers. The genomic analysis revealed that all strains had the metabolic potential of synthesizing PHAs, except for strain NTK071, which seemed to be missing the PHA synthase gene (*phaC*) required for polymerizing PHA monomers. However, we did not find intracellular PHA depolymerases nor extracellular PHA depolymerases or hydrolases in our annotated genomes. This came as a surprise, since we expected depolymerases or hydrolases for breaking down (extracellular) forms of PHAs in our consortium members. The sole exception was *Rhodobacter* sp. NTK016 that had a predicted PhaZ, albeit intracellular. The PhaZ depolymerases were, however, correctly annotated in *B. infantis* NRRL B-14911¸ known to have two depolymerases according to the PHA Depolymerase Engineering Database [42]: one experimentally validated extracellular PhaZ [118] and one intracellular depolymerase, often annotated as 3-oxoadipate enol-lactonase. Furthermore, the depolymerases in *R. sphaeroides* 2.4.1 that were deposited in the database were correctly annotated. However, one of the sequences deposited in the database as an extracellular depolymerase, we predicted with SignalP 4.1, does not contain a signal peptide. These known depolymerases also did not cluster together in Anvi’o with genes from our own strains.

PhaZ genes are not well conserved at the nucleotide level [42], however amino acid conservation is often observed for the oxyanion hole, lipase box, and catalytic domain motifs. These features also occur in other protein members of the alpha/beta hydrolase superfamily, like esterases and lipases [38]. We therefore searched for other depolymerizing/hydrolyzing candidates in the genomes of the two PHA-plate clearing isolates *Bacillus* sp. NTK074B and *V. proteolyticus* NBRC 13287. We filtered for extracellular proteins and performed domain-based searches for alpha/beta hydrolases. We detected several candidate proteins for both strains and after using a motif search for the oxyanion hole and lipase box, we honed in on a single candidate for both of the strains. The remaining amino acids of the catalytic triad, aspartic acid and histidine, could also be identified in these candidates. In *Bacillus* sp. NTK074B the candidate gene was annotated as a hypothetical protein and in *V. proteolyticus* NBRC 13287, as a lipase precursor (EC 3.1.1.3). Based on the gene clustering from Anvi’o, the former protein was conserved in close relatives, but not in other NTK isolates. The latter protein was conserved in most of the close relatives, and also in *V. alginolyticus* ATCC 33787 (85.2% amino acid sequence similarity), however, this strain did not show clearing zones. The order of the conserved features in all the sequences was: (1) oxyanion hole; (2) lipase box with serine of the catalytic triad; and, (3) remaining amino acids of the catalytic triad: aspartic acid and histidine. In the PHA Depolymerase Engineering Database [42], PhaZ7 from *Paucimons lemoignei* was the most similar to our candidates, since it also contained the same order of conserved features. This enzyme was experimentally validated as an extracellular depolymerase [85].

A recent biodegradation study similarly reported a lack of depolymerases but presence of lipases, working with a strain of *Acinetobacter lwoffii* capable of producing clearing zones on PHA plates [86]. This bacterium also grew in liquid cultures with PHAs as the sole carbon source. These authors further performed lipase activity tests with secreted enzymes from the strain, which confirmed lipase activity. When analyzing two available genomes of strains of *A. lwoffii*, they found no genes encoding intracellular or extracellular PHA depolymerases. However, they did find 3–4 genes encoding secretory lipases, also similar to PhaZ7 of *P. lemoignei*. Based on its amino acid sequence, PhaZ7 from *P. lemoignei* groups together with triacylgrylerol lipases (EC 3.1.1.3), within the carboxylic ester hydrolases [41]. The EC-number of the triacylglycerol lipases matched that of our candidate depolymerase from *V. proteolyticus*, the lipase precursor. The closely related carboxylic ester group members also include secreted triacylglycerol lipases from *Burkholderia cepacia*, *Burkholderia glumae* (formerly *Chromobacterium viscosum*) and *Pseudomonas aeruginosa*, a thermostable lipase from *Geobacillus zalihae* and lipase A from *Bacillus subtilis*. A structural comparison of the PhaZ7 protein with other protein structures in the Protein Data Bank (PDB) [119] showed a high level of similarity with lipase A from *Bacillus subtilis* [93]. Extracellular lipases from these organisms have in fact been reported to hydrolyze some forms of PHA. Extracellular lipases from *B. cepacia*, *B. subtilis*, and *P. aeruginosa* were reported to hydrolyze poly(4-hydroxybutyrate), but not poly(3-hydroxybutyrate) and poly(3-hydroxyvalerate). These species were also not able to hydrolyze the co-polymers poly(3-hydryxoybutyrate-co-3-hydroxyhexanoate-co-3-hydroxyoctanoate), poly(3-hydroxyhexanoate-co-3-hydroxyoctanoate) and poly(3-hydroxyhexanoate-co-3-hydroxyoctanote-co-3-hydroxydecanoate-co-3-hydroxydodecanoate), with the exception of *P. aeruginosa*, which could hydrolyze all three [120]. Furthermore, a commercial lipase from *Burkholderia glumae* was tested and confirmed to hydrolyze poly(3-hydroxypropionate), but not poly(3-hydroxybutyrate), poly(4-hydroxybutyrate), poly(5-hydroxyvalerate) and poly(6-hydroxyhexanoate) [121]. Most-recently it was proven that the thermostable lipase from *Geobacillus zalihae* T1 (GenBank accession: Q842J9) had amorphous poly(3-hydroxybutyrate) degradation activity as well [122].

When it comes to the actual utilization of the PHA monomers, the essential genes for oxidation and CoA hydrolase/transferase were present in all the isolates, except for the *Vibrio* strains. None of the analyzed *Vibrio* genomes contained the *bdh* gene, responsible for encoding a protein converting hydroxyacyl monomers to oxo acids. To the best of our knowledge, this gene does not occur in *Vibrio* spp. in general. Assuming that the original identity of the NTK045 and NTK049 strains was correct, this coincides with the inability to grow on PHAs as a sole carbon source, while we did find potential extracellular depolymerases in the genomes of *V. proteolyticus* NBRC 13287 and *V. alginolyticus* ATCC33787. All of the analyzed *Vibrio* genomes do, however, contain an annotated gene encoding *Hibdh* (3-hydroxyisobutyrate dehydrogenase, EC 1.1.1.31), that converts 3-hydroxy-2-methylpropanoate (3-hydroxyisobutyrate) to 2-methyl-3-oxopropanoate. Although *Hibdh* does not convert (R)-3-hydroxybutyrate, this enzyme can convert 3-hydroxypropanoate in *Bacillus cereus* [123,124], *Pseudomonas denitrificans* [125,126], and *Pseudomonas putida* [127]. Potentially, the vibrios in the consortium can utilize depolymerized 3-carbon-length hydroxyalkanoate monomers, although the KM value (Michaelis–Menten constant) with 3-hydroxypropanoate is at least a factor 10 to 20 times higher than with its natural substrate in the aforementioned species.

## 5. Conclusions

In the present study, we employed DNA marker gene and genome sequencing to ascertain the identities of nine members of an experimentally-confirmed PHA biodegrading bacterial consortium. These members belonged to the *Bacillaceae*, *Rhodobacteraceae*, and *Vibrionaceae* families. Of the consortium, only two individual members showed the ability to utilize PHAs as a sole carbon substrate. One of these two isolates and two others showed depolymerase activity in a clearing-zone assay. This could indicate a synergy of the consortium members when the full consortium was employed to biodegrade PHAs. Genomes for two of the members were already available and we sequenced genomes of four more members. Using comparative genomics, we found that most of the members have necessary enzymes for both PHA production and utilization, however, we did not find classical PHA depolymerases or hydrolases. We did identify two potentially new extracellular depolymerases in two of the three consortium members that showed depolymerase activity. These enzymes resemble triacylglycerol lipases and have the required catalytic triad, oxyanion hole, and lipase box to function as external PHA depolymerases. Our findings coincide with a recent study [86], that lends credence to the existence of alternative enzymes that may degrade PHAs externally in nature, expanding the possible repertoire of enzymes capable of doing so, with lipases. This has real-world implications and presents caveats for studies that only considered conventional PHA depolymerase signatures in environmental and plastic colonization studies.

## Figures and Tables

**Table 1:** Identities of the biodegradation community isolates, as determined by the original identification methods, isolate fate since original biodegradation testing, and the isolate identity as determined by the sequenced 16S rRNA gene sequence.

Isolate	Original Identity	Isolate Fate	16S rRNA Gene BLASTn Hit	Coverage	Identity	Accession
NTK009	Unknown	Lost	-	-	-	-
NTK016B	Unknown	Revived	*Rhodobacter* sp. R18	99.72%	97%	AB607872
NTK029	*Pseudoalteromonas haloplanktis*	Revived	*Bacillus cereus* group	100%	99.93%	-
NTK034	Unknown	Revived	*Bacillus oceanisediminis* 2691	100%	99.87%	CP015506
NTK039	*Pseudomonas creosotensis*	Lost	-	-	-	-
NTK045	*Vibrio proteolyticus*	Replaced by ATCC 15338	*Vibrio proteolyticus* ATCC 15338 = NBRC 13287	100%	99.64%	NR_026128
NTK049	*Vibrio alginolyticus*	Replaced by ATCC 33787	*Vibrio alginolyticus* ATCC 33787	100%	99.35%	CP013484
NTK060	*Vibrio campbellii*	Lost	-	-	-	-
NTK071	*Bacillus megaterium*	Revived	*Bacillus* sp. N1-1	100%	99.49%	CP046564
NTK072	*Vibrio furnissii*	Revived	*Bacillus atrophaeus*, multiple strains	100%	100%	-
NTK073	*Xanthomas campestris*	Lost	-	-	-	-
NTK074Act	*Actinomycete* sp.	Lost	-	-	-	-
NTK074B	Unknown	Revived	*Bacillus vietnamensis* 151-6	100%	99.68%	CP047394
NTK_Randy	*Bacillus* sp.	Revived	*Bacillus cereus* group	100%	99.93%	-

**Table 2:** Growth results of the NTK isolates in liquid medium with PHAs as the sole carbon source and determination of depolymerase activity, as assessed by clearing zones formed after 2 months on PHA covered culture plates. Genome availability is indicated in the last column. N.D. = not determined.

Isolate	16S rRNA Gene Top Blastn Hit	Growth on PHAs	Depolymerase Activity	Sequenced Genome
ATCC 15338	*V. proteolyticus* ATCC 15338 = NBRC 13287	N.D. **^a^**	Yes	Existing
ATCC 33787	*V. alginolyticus* ATCC 33787	N.D. **^a^**	No	Existing
NTK016B	*Rhodobacter* sp. R18	No	No	This study
NTK029	*B. cereus* group	No	N.D.	No
NTK034	*B. oceanisediminis* 2691	No	No	This study
NTK071	*Bacillus* sp. N1-1	No	No	This study
NTK072	*B. atrophaeus*, multiple strains	No	Yes	No
NTK074B	*B. vietnamensis* 151-6	Yes	Yes	This study
NTK_Randy	*B. cereus* group	Yes	No	No

**^a^** The original NTK isolates NTK045 and NTK049, did not show growth on PHAs.

**Table 3:** Genome features of sequenced NTK isolates.

Attribute	NTK016B	NTK034	NTK071	NTK074B
Genome size (bp)	4,854,159	5,599,963	4,164,462	4,250,699
GC-content (%)	65.4	41.0	39.9	43.5
Number of contigs	162	100	229	446
N50	231,698	423,522	225,829	300,122
L50	8	4	5	5
Average coverage (reads)	63.8	51.8	51.7	74.0
Number of coding sequences	4889	6087	4385	4852
Number of RNAs	46	127	102	128

**Table 4:** Presence (✓) and absence (-) overview of PHA biosynthesis- and degradation-related genes in the annotated genomes, based on the functional annotation.

	Function	1	2	3	4	5	6	7	8	9	10	11	12
Species	Gene	*phaA*/*bktB*	*fabG*	*fadB*	*hbd*	*phaB*	*phaC*	(e) *phaY*	(e) *phaZ*	(i) *phaZ*	*bdh*	*aacS*	*scoA* and *scoB*
*Bacillus* sp. NTK034 ◄		✓	✓	✓	✓	-	✓	-	-	-	✓	-	✓
*Bacillus oceanisediminis* 2691		✓	✓	✓	✓	-	✓	-	-	-	✓	-	✓
*Bacillus oceanisediminis* H2		✓	✓	✓	✓	-	✓	-	-	-	✓	-	✓
*Bacillus infantis* NRRL B-14911		✓	✓	✓	✓	-	✓	-	✓	✓	✓	-	✓
*Bacillus firmus* NCTC 10335		✓	✓	✓	✓	-	✓	-	-	-	✓	-	✓
*Bacillus vietnamensis* NBRC 101237		✓	✓	✓	✓	-	✓	-	-	✓	✓	-	✓
*Bacillus vietnamensis* 151-6		✓	✓	✓	✓	-	✓	-	-	✓	✓	-	✓
*Bacillus* sp. NTK074B ◄		✓	✓	✓	✓	-	✓	-	-	-	✓	-	✓
*Bacillus aquimaris* TF-12		✓	✓	✓	✓	-	-	-	-	✓	✓	-	✓
*Bacillus marisflavi* TF-11		✓	✓	✓	✓	-	✓	-	-	-	✓	-	✓
*Bacillus* sp. NTK071 ◄		✓	✓	✓	✓	-	-	-	-	-	✓	-	✓
*Bacillus hwajinpoensis* Y2		✓	✓	✓	✓	-	-	-	-	-	✓	-	✓
*Anaerobacillus macyae* DSM 16346		✓	✓	✓	✓	-	-	-	✓	✓	✓	-	✓
*Bacillus* sp. N1-1		✓	✓	✓	✓	-	-	-	-	-	✓	-	✓
*Bacillus hwajinpoensis* 22506_14_FS		✓	✓	✓	✓	-	-	-	-	-	✓	-	✓
*Rhodobacter sphaeroides* 2.4.1		✓	✓	✓	✓	✓	✓	-	✓ **^a^**	✓	✓	-	✓
*Defluviimonas alba* cai42		✓	✓	✓	✓	✓	✓	-	-	✓	✓	-	✓
*Roseicitreum antarcticum* ZS2-28		✓	✓	✓	✓	✓	✓	-	-	✓	✓	-	✓
*Rhodobacter* sp. NTK016B ◄		✓	✓	✓	✓	✓	✓	-	-	✓	✓	✓	✓
*Pararhodobacter* sp. CIC4N-9		✓	✓	✓	✓	✓	✓	-	-	✓	✓	✓	✓
*Pararhodobacter* sp. CCB-MM2		✓	✓	✓	✓	✓	✓	-	-	✓	✓	✓	✓
*Vibrio furnissii* ATCC 35016		✓	✓	✓	-	✓	✓	-	-	-	-	✓	-
*Vibrio proteolyticus* NBRC 13287 ◄		✓	✓	✓	-	✓	✓	-	-	-	-	✓	-
*Vibrio tubiashii* ATCC 19109		✓	✓	✓	-	✓	✓	-	-	-	-	✓	-
*Vibrio atypicus* HHS02		✓	✓	✓	✓	✓	✓	-	-	-	-	✓	-
*Vibrio parahaemolyticus* ATCC 17802		✓	✓	✓	-	✓	✓	-	-	-	-	✓	-
*Vibrio diabolicus* FDAARGOS_105		✓	✓	✓	-	✓	✓	-	-	-	-	✓	-
*Vibrio alginolyticus* NBRC 15630		✓	✓	✓	-	✓	✓	-	-	-	-	✓	-
*Vibrio alginolyticus* ATCC 33787 ◄		✓	✓	✓	-	✓	✓	-	-	-	-	✓	-
*Vibrio natriegens* NBRC 15636		✓	✓	✓	-	✓	✓	✓	-	-	-	✓	-
*Vibrio rotiferianus* B64D1		✓	✓	✓	-	✓	✓	-	-	-	-	✓	-
*Vibrio harveyi* FDAARGOS_107		✓	✓	✓	-	✓	✓	-	-	-	-	✓	-
*Vibrio campbellii* CAIM 519		✓	✓	✓	-	✓	✓	-	-	-	-	✓	-
**Function:**													
**1**	Acetyl-CoA acetyltransferase/β-ketothiolase (EC 2.3.1.9 and EC 2.3.1.16)				**7**	(extracellular) PHA oligomer hydrolase (EC 3.1.1.22)							
**2**	3-oxoacyl-[acyl-carrier-protein] reductase (EC 1.1.1.100 with EC 1.1.1.36 capacity)				**8**	(extracellular) PHA depolymerase (EC 3.1.1.75 and EC 3.1.1.76)							
**3**	Enoyl-CoA hydratase (EC 4.2.1.17)/ Hydroxyacyl-CoA dehydrogenase (EC 1.1.1.35)				**9**	(intracellular) PHA depolymerase (EC 3.1.1.75 and EC 3.1.1.76)							
**4**	3-hydroxybutyryl-CoA dehydrogenase (EC 1.1.1.157)				**10**	3-hydroxybutyrate dehydrogenase (EC 1.1.1.30)							
**5**	Acetoacetyl-CoA reductase (EC 1.1.1.36)				**11**	Acetoacetyl-CoA synthetase (EC 6.2.1.16)							
**6**	PHA synthase (EC 2.3.1.-)				**12**	3-oxoacid CoA-transferase subunit A and B (EC 2.8.3.5)							

◄ NTK biodegradation consortium member. **^a^** Deposited in the PHA Depolymerase Engineering Database [42] as an extracellular depolymerase, but contains no signal peptide, according to SignalP 4.1.

## Data Availability

The 16S rRNA gene sequence data presented in this study are publicaly available in GenBank. The 16S rRNA gene sequences obtained in this study can be found there under accession numbers MW435594-MW435596. The genome sequence data presented in this study are publicaly available at NCBI. The genome data obtained in this study can be found there under BioProject number PRJNA649735. More specifically, the raw genome sequence reads are deposited in the NCBI Sequence Read Archive with accession numbers SRR12354198-SRR12354201 and annotated genomes are deposited in INSDC under accession numbers CPXXXXXX-CPXXXXXX. The annotated genomes are also available online at RAST via https://rast.nmpdr.org/, when logging in with a guest-account. They can be found under ID numbers 6666666.521526, 6666666.521528, 6666666.521530 and 6666666.521531. Extracellular PHA depolymerases and closely related lipases used for comparison with our putative PHA depolymerase candidates, are publicaly available in NCBI Protein.

## Supplementary Materials

The following are available online at https://www.mdpi.com/2076-2607/9/1/186/s1, Table S1: Metadata regarding species used for the phylogenetic analysis of the NTK community strains. Strain synonyms, isolation-associated information, and type strain designation are mentioned. Table S2: 16S rRNA gene sequence information for the taxa included in phylogenetic tree reconstruction. Table S3: Genome availability of species in NCBI used for phylogenetic analysis and comparative genomics. Included are the GenBank accession number, assembly level, and representative genome status for a given species. Table S4: Properties of the genomes used for the *Bacillaceae* comparative genomics analysis with Anvi’o. Table S5: Pangenomic analysis properties of the *Rhodobacteraceae* genomes that were compared with NTK community NTK016B. Table S6: Properties of genomes used for the *Vibrionaceae* pangenomic analysis with the NTK community strains. Table S7: Amino acid sequences of the extracellular PHA depolymerase candidates. Table S8: Positions of conserved sites in known extracellular PHA depolymerases and closely related lipases, with candidate extracellular PhaZs. Table S9: MiXS table with metadata for sequenced NTK isolate rRNA marker genes (MIMARKS). Table S10: MiXS tables with metadata for sequenced NTK isolate genomes (MIGS).
