# Correction: De Vogel, F.A., et al. Comparative Genomics of Marine Bacteria from a Historically Defined Plastic Biodegradation Consortium with the Capacity to Biodegrade Polyhydroxyalkanoates. *Microorganisms* 2021, *9*, 186

**DOI:** 10.3390/microorganisms9040744

**Published:** 2021-04-02

**Authors:** Fons A. de Vogel, Cathleen Schlundt, Robert E. Stote, Jo Ann Ratto, Linda A. Amaral-Zettler

**Affiliations:** 1 Department of Marine Microbiology and Biogeochemistry, NIOZ Royal Netherlands Institute for Sea Research, P.O. Box 59, 1790 AB Den Burg, The Netherlands; fons.de.vogel@nioz.nl; 2 Faculty of Geosciences, Department of Earth Sciences, Utrecht University, P.O. Box 80.115, 3508 TC Utrecht, The Netherlands; 3 Josephine Bay Paul Center for Comparative Molecular Biology and Evolution, Marine Biological Laboratory, Woods Hole, MA 02543, USA; cschlundt@geomar.de; 4 U.S. Army Combat Capabilities Development Command Soldier Center, 10 General Greene Avenue, Natick, MA 01760, USA; robert.e.stote.civ@mail.mil (R.E.S.); joann.rattoross.civ@mail.mil (J.A.R.); 5 Department of Freshwater and Marine Ecology, Institute for Biodiversity and Ecosystem Dynamics, University of Amsterdam, P.O. Box 94240, 1090 GE Amsterdam, The Netherlands

The authors wish to make the following corrections to this paper [1]:

## 1. Change to Section 3.1. Isolate Identification

The 16S rRNA gene sequences are deposited under GenBank accession numbers MW435594-MW435596 and in CPXXXXXXX-CPXXXXX. Associated metadata are specified in Supplementary Tables S9 and S10.

To the correct version, as follows:

The 16S rRNA gene sequences are deposited under GenBank accession numbers MW435594-MW435596 and in JAFKOK000000000, JAFKOJ000000000, JAFKOI000000000, and JAFKOH000000000. Associated metadata are specified in Supplementary Tables S9 and S10.

## 2. Change to Section 3.4. Whole-Genome Sequencing

The RASTtk annotated genomes are available online via https://rast.nmpdr.org/ by logging in as guest and can be found under ID numbers 6666666.521526 (Rhodobacter sp. NTK016B), 6666666.521528 (*Bacillus* sp. NTK034), 6666666.521530 (*Bacillus* sp. NTK071) and 6666666.521531 (*Bacillus* sp. NTK074B). The raw sequence reads are deposited in the NCBI Sequence Read Archive under BioProject PRJNA649735 with sample accession numbers SRR12354198 to SRR12354201. Annotated genomes are also deposited in INSDC under references CPXXXXXX-CPXXXXXX, and associated metadata are specified in Supplementary Table S10.

To the correct version, as follows:

The RASTtk annotated genomes are available online via https://rast.nmpdr.org/ by logging in as guest and can be found under ID numbers 6666666.521526 (*Rhodobacter* sp. NTK016B), 6666666.521528 (*Bacillus* sp. NTK034), 6666666.521530 (*Bacillus* sp. NTK071), and 6666666.521531 (*Bacillus* sp. NTK074B). The raw sequence reads are deposited in the NCBI Sequence Read Archive under BioProject PRJNA649735 with sample accession numbers SRR12354198 to SRR12354201. Assembled genomes are deposited in INSDC under references JAFKOK000000000, JAFKOJ000000000, JAFKOI000000000, and JAFKOH000000000, and associated metadata are specified in Supplementary Table S10.

## 3. Change to Data Availability Statement

The genome data obtained in this study can be found there under BioProject number PRJNA649735. More specifically, the raw genome sequence reads are deposited in the NCBI Sequence Read Archive with accession numbers SRR12354198-SRR12354201 and annotated genomes are deposited in INSDC under accession numbers CPXXXXXX-CPXXXXXX. The annotated genomes are also available online at RAST via https://rast.nmpdr.org/, when logging in with a guest-account. They can be found under ID numbers 6666666.521526, 6666666.521528, 6666666.521530, and 6666666.521531.

To the correct version, as follows:

The genome data obtained in this study can be found there under BioProject number PRJNA649735. More specifically, the raw genome sequence reads are deposited in the NCBI Sequence Read Archive with accession numbers SRR12354198-SRR12354201 and assembled genomes are deposited in INSDC under accession numbers JAFKOK000000000, JAFKOJ000000000, JAFKOI000000000, and JAFKOH000000000. The annotated genomes are available online at RAST via https://rast.nmpdr.org/, when logging in with a guest-account. They can be found under ID numbers 6666666.521526, 6666666.521528, 6666666.521530, and 6666666.521531.

## 4. Addition to Supplementary Table S8


**Protein**

**NCBI Protein Accession**

**Species**

**Signal Peptide**

**Oxyanion Hole**

**Lipase Box**

**Catalytic Triad (S-H-A)**

**Reference**
Hypothetical protein ◄
XXXXXX
*Bacillus* sp. NTK074B1-2257-57119-123121-223-256This studyHypothetical protein sed_3530ABV38134*Shewanella sediminis* HAW-EB31-2367-73160-164162-269-331[84]
**Lipase A**

**P37957**

***Bacillus subtilis***
**strain 168**

**1-31**

**36-42**

**106-110**

**108-164-187**
[85]Lipase class 2ABZ77296*Shewanella halifaxensis* HAW-EB41-2367-73160-164162-269-331[84]Lipase precursor ◄
XXXXXX
*Vibrio alginolyticus* ATCC 337871-2333-39104-108106-253-275
This study
Lipase precursor ◄
XXXXXX
*Vibrio proteolyticus* NBRC 132871-2335-41106-110108-255-277
This study

**PhaZ7**

**Q939Q9**

***Paucimonas lemoignei***

**1-38**

**80-86**

**172-176**

**174-280-344**
[86–88]
**Thermostable lipase**

**Q842J9**

***Geobacillus zalihae***
**T1**

**1-28**

**37-43**

**139-143**

**141-345-386**
[89,90]Triacylglycerol lipaseWP_005101273*Acinetobacter* multispecies1-2950-56125-129127-280-302[91]Triacylglycerol lipaseWP_005101276*Acinetobacter* multispecies1-2344-50120-124122-267-289[91]Triacylglycerol lipaseWP_064094572*Acinetobacter* multispecies1-2142-48117-121119-266-288[91]
**Triacylglycerol lipase**

**P22088**

***Burkholderia cepacian***
**ATCC 21808**

**1-44**

**54-60**

**129-133**

**131-308-330**
[92,93]
**Triacylglycerol lipase**

**Q05489**

***Burkholderia glumae***
**ATCC 6918**

**1-39**

**49-55**

**124-128**

**126-302-324**
[94,95]
**Triacylglycerol lipase**

**P26876**

***Pseudomonas aeruginosa***
**PAO1**

**1-26**

**35-41**

**106-110**

**108-255-277**
[96,97]

To the correct version, as follows:


**Protein**

**NCBI Protein Accession**

**Species**

**Signal Peptide**

**Oxyanion Hole**

**Lipase Box**

**Catalytic Triad (S-H-A)**

**Reference**
Hypothetical protein ◄
MBN8191285
*Bacillus* sp. NTK074B1-2257-57119-123121-223-256This studyHypothetical protein sed_3530ABV38134*Shewanella sediminis* HAW-EB31-2367-73160-164162-269-331[84]
**Lipase A**

**P37957**

***Bacillus subtilis***
**strain 168**

**1-31**

**36-42**

**106-110**

**108-164-187**
[85]Lipase class 2ABZ77296*Shewanella halifaxensis* HAW-EB41-2367-73160-164162-269-331[84]Lipase precursor ◄
ALR91050
*Vibrio alginolyticus* ATCC 337871-2333-39104-108106-253-275
Direct Submission (08-DEC-2015)
Lipase precursor ◄
GAD66740
*Vibrio proteolyticus* NBRC 132871-2335-41106-110108-255-277
Direct submission (10-SEP-2013)

**PhaZ7**

**Q939Q9**

***Paucimonas lemoignei***

**1-38**

**80-86**

**172-176**

**174-280-344**
[86–88]
**Thermostable**
**lipase**

**Q842J9**

***Geobacillus zalihae***
**T1**

**1-28**

**37-43**

**139-143**

**141-345-386**
[89,90]Triacylglycerol lipaseWP_005101273*Acinetobacter* multispecies1-2950-56125-129127-280-302[91]Triacylglycerol lipaseWP_005101276*Acinetobacter* multispecies1-2344-50120-124122-267-289[91]Triacylglycerol lipaseWP_064094572*Acinetobacter* multispecies1-2142-48117-121119-266-288[91]
**Triacylglycerol**
**lipase**

**P22088**

***Burkholderia cepacian***
**ATCC 21808**

**1-44**

**54-60**

**129-133**

**131-308-330**
[92,93]
**Triacylglycerol**
**lipase**

**Q05489**

***Burkholderia glumae***
**ATCC 6918**

**1-39**

**49-55**

**124-128**

**126-302-324**
[94,95]
**Triacylglycerol**
**lipase**

**P26876**

***Pseudomonas aeruginosa***
**PAO1**

**1-26**

**35-41**

**106-110**

**108-255-277**
[96,97]

## 5. Addition to Table S10 (only Partly Shown):


**NCBI BioProject number**

**PRJNA649735**

**PRJNA649735**

**PRJNA649735**

**PRJNA649735**

**NCBI BioSample number**

**SAMN15677905**

**SAMN15677906**

**SAMN15677907**

**SAMN15677908**

**NCBI SRA accession number**

**SRR12354201**

**SRR12354200**

**SRR12354199**

**SRR12354198**

**NCBI Genome accession number**






To the correct version, as follows:
**NCBI BioProject number****PRJNA649735****PRJNA649735****PRJNA649735****PRJNA649735****NCBI BioSample number****SAMN15677905****SAMN15677906****SAMN15677907****SAMN15677908****NCBI SRA accession number****SRR12354201****SRR12354200****SRR12354199****SRR12354198****NCBI Genome accession number****JAFKOK000000000****JAFKOJ000000000****JAFKOI000000000****JAFKOH000000000**

The authors would like to apologize to the readers for any inconvenience caused by these changes.

## References

[1] De Vogel F.A., Schlundt C., Stote R.E., Ratto J.A., Amaral-Zettler L.A. (2021). Comparative Genomics of Marine Bacteria from a Historically Defined Plastic Biodegradation Consortium with the Capacity to Biodegrade Polyhydroxyalkanoates. Microorganisms.

