# Supplementary material for: Comparative Genomics of Marine Bacteria from a Historically Defined Plastic Biodegradation Consortium with the Capacity to Biodegrade Polyhydroxyalkanoates
Source: Microorganisms. 2021 Jan 16;9(1):186. doi: 10.3390/microorganisms9010186 (PMC7830162; doi:10.3390/microorganisms9010186)
Supplement: Supplementary file 1 [file microorganisms-09-00186-s001.pdf]

Supplementary Materials

Table S1. Metadata regarding species used for the phylogenetic analysis of the NTK community strains. Strain synonyms, isolation-associated information, and type strain designation are mentioned.

Species	Strain synonyms	Isolation associated information	Type strain	Reference / GenBank accession
Aliivibrio fischeri ATCC 7744	BCRC 12900, CAIM 329, CCUG 13450, CCUG 16305, CECT 524, CIP 103206, CIP 75.08, DSM 507, LMG 4414, JCM 18803, KCTC 12272, KCTC 12386, NCIMB 1281, NRRL B-761, NRRL B-41164	Isolation source: fish (bioluminescent)<br>Collection date: 1888<br>Location: N/A	✓	[1]
Anaerobacillus macyae DSM 16346	CIP 108766, JCM 12340, JMM-4	Isolation source: gold mine<br>Collection date: 01-JAN-2004<br>Location: Bendigo, Victoria, Australia	✓	[2]
Bacillus algicola KMM 3737	CIP 107850, KCTC 13005	Isolation source: *Fucus evanescens* (brown algae)<br>Collection date:1999<br>Location: Kraternaya Bight, Kuril Islands	✓	[3]
Bacillus altitudinis 41KF2b	BCRC 17879, DSM 21631, JCM 13350, MTCC 7306	Isolation source: cryogenic tube used for collecting air samples<br>Collection date: 2001<br>Location: 41 km altitude, India (17.4722°/ 78.5800°)	✓	[4]
Bacillus amyloliquefaciens DSM 7	ATCC 23350, BCRC 11601, Campbell F, CCUG 28519, F, Fukomoto F, IFO 15535, NBRC 15535, NCCB 91058, NRRL B-14393	Isolation source: soil and industrial amylase fermentations<br>Collection date: before 1943<br>Location: N/A	✓	[5]
Bacillus aquimaris TF-12	DSM 16205, JCM 11545, KCCM 41589	Isolation source: sea water of a tidal flat<br>Collection date: 06-NOV-2009<br>Location: Yellow Sea, South Korea	✓	[6]
Bacillus atrophaeus 1942	Vogel	Isolation source: unknown, terrestrial<br>Collection date: 1930s-1940s presumably<br>Location: N/A	-	[7]
Bacillus atrophaeus JCM9070	ATCC 49337, BCRC 17123, CCUG 28524, DSM 7264, NBRC 15539, NCIMB 12899, NRRL-NRS 213	Isolation source: soil<br>Collection date: 1940<br>Location: Colorado, USA	✓	[8]
Bacillus cereus ATCC 14579	BCRC 10603, CCM 2010, CCUG 7414, CIP 66.24, DSM 31, Ford 13, Gibson 971, HAMBI 1887, HAMBI 1905, IAM 12605, JCM 2152, LMG 6923, NBRC 15305, NCCB 75008, NCIMB 9373, NCTC 2599, NRRL B-3711, UCM B-5650, VKM B-504	Isolation source: air<br>Collection date: before 1887<br>Location: N/A	✓	[9]
Bacillus cytotoxicus NVH 391-98	CIP 110041, DSM 22905	Isolation source: vegetable puree, related to food poisoning<br>Collection date: 1998<br>Location: retirement house, France	✓	[10]

Bacillus firmus EN 5C	N/A	Isolation source: agricultural soil Collection date: N/A Location: Visakhapatnam district, Andhra Pradesh, India	-	[11]
Bacillus firmus NCTC 10335	ATCC 14575, BCRC 11730, CCM 2213, CCUG 7418, CIP 52.70, DSM 12, HAMBI 1886, IAM 12464, JCM 2512, LMG 7125, NBRC 15306, NCAIM B.01087, NCCB 48015, NCIMB 9366, NCTC 10335, NRRL B-14307, NRRL NRS-613, VKM B-498	Isolation source: soil Collection date: before 1933 Location: N/A	✓	[12]
Bacillus gottheilii WCC 4585	CCUG 59876, DSM 23668, G 6155, LMG 25856	Isolation source: clean room of production line of pharmaceutical manufacturing site for vaccines Collection date: 2008 Location: Sachsen-Anhalt, Dessau, Germany	✓	[13]
Bacillus hwajinpoensis 22506_14_FS	N/A	Isolation source: fleur de sel salt Collection date: N/A Location: grocery store, France	-	WMEY01000019
Bacillus hwajinpoensis Y2	N/A	Isolation source: salt water Collection date: 01-Feb-2019 Location: Hampton Beach, New Hampshire, USA (42.899 N / 70.81085 W)	-	SWFM01000018
Bacillus infantis NRRL B-14911	N/A	Isolation source: open ocean 10 m depth Collection date: JUN-1992 Location: Gulf of Mexico, near the Bimini Islands, Bahamas	-	[14]
Bacillus krulwichiae AM31D	DSM 18225, IAM 15000, JCM 11691, NCIMB 13904	Isolation source: aromatic-compound-contaminated garden soil Collection date: before 2003 Location: Tsukuba, Ibaraki, Japan (36.1167 N/ 140.2167 E)	✓	[15]
Bacillus marisflavi 151-25	N/A	Isolation source: Cd-contaminated soil (59.561 ± 3.76 mg Cd kg^{-1}) Collection date: APR-2016 Location: Hunan, China	-	[16]
Bacillus marisflavi TF-11	DSM 16204, JCM 11544, KCCM 41588	Isolation source: sea water of a tidal flat Collection date: 01-JAN-2003 Location: Yellow Sea, South Korea	✓	[6]
Bacillus megaterium IAM 13418	ATCC 14581, BCRC 10608, CCM 2007, CCUG 1817, CIP 66.20, DSM 32, Ford 19, Gibson 1060, HAMBI 2018, JCM 2506, KCTC 3007, LMG 7127, NBRC 15308, NCCB 75016, NCIMB 9376, NCTC 10342, NRIC 1710, NRRL B-14308, VKM B-512	Isolation source: environmental sample Collection date: before 1884 Location: N/A	✓	[17]
Bacillus oceanisediminis 2691	KCTC 33020	Isolation source: marine sediment Collection date: AUG-1999 Location: Tae-an-gun, Chungchungnam-do, South Korea	-	[18]

Bacillus oceanisediminis H2	CGMCC 1.10115, DSM 24771, JCM 16506	Isolation source: marine sediment, 823 m depth<br>Collection date: before 2010<br>Location: South Sea, China	✓	[19]
Bacillus oryzaecorticis IHB B 17121	N/A	Isolation source: saffron rhizosphere<br>Collection date: before 2017<br>Location: Drussu, Jammu & Kashmir, India	-	KY933462
Bacillus pseudofirmus DSM 8715	ATCC 700159, C 324, NCIMB 10283, PN 3	Isolation source: lake bank soil<br>Collection date: before 1994<br>Location: Holstein, Germany	✓	[20]
Bacillus shackletonii LMG 18435	B1724, DSM 18868, CIP 107762, KCTC 13003	Isolation source: mossy soil, eastern lava flow<br>Collection date: 1996–1997 austral summer<br>Location: Lucifer Hill, northern Candlemas Island, South Sandwich archipelago	✓	[21]
Bacillus sp. BW3PhG2	N/A	Isolation source: *Pyropia haitanensis* (red algae) phycosphere<br>Collection date: NOV-2010<br>Location: China	-	[22]
Bacillus sp. JL-29	N/A	Isolation source: eutrophic estuary<br>Collection date: before 2006<br>Location: Yangtze River, China	-	[23]
Bacillus sp. MN-003	N/A	Isolation source: tropical marine sediments contaminated with marine fuel oil<br>Collection date: before 2002<br>Location: beach in south Singapore	-	[24]
Bacillus sp. N1-1	N/A	Isolation source: deep-sea water, 1133 m depth<br>Collection date: 24-SEP-2017<br>Location: South China Sea, China (22.115 N 119.283 E)	-	[25]
Bacillus subtilis DSM10	ATCC 6051, BGSC 3A1, CCM 2216, CCUG 163, IAM 12118, IFO 13719, JCM 1465, LMG 7135, NBRC 13719, NCIB 3610, NCTC 3610, NCDO 1769, NCIMB 3610, NRRL NRS-744, Marburg	Isolation source: N/A<br>Collection date: around 1900<br>Location: N/A	✓	[26]
Bacillus thuringiensis ATCC 10792	Berliner, CCM 19, CCUG 7429, CIP 53.137, DSM 2046, HAMBI 478, IAM 12077, LMG 7138, NCAIM B.01292, NCCB 70008, NCIB 9134, NRRL HD-735, VKM B-1544	Isolation source: *Ephestia kuhniella* (Mediterranean flour moth)<br>Collection date: before 1915<br>Location: N/A	✓	[27]
Bacillus toyonensis BCT-7112	NCIMB 14858, CECT 876	Isolation source: soil<br>Collection date: 01-JAN-1966<br>Location: Izu, Shizuoka, Japan	✓	[28]

Bacillus vietnamensis 151-6	N/A	Isolation source: Cd-contaminated soil (59.561 ± 3.76 mg Cd kg^{-1}) Collection date: APR-2016 Location: Hunan, China	-	[16]
Bacillus vietnamensis NBRC 101237	BCRC 80102, CIP 108672, DSM 18898, HSCC 1663, JCM 11124, NRIC 0531, NRRL B-23890, LMG 24742, 15-1	Isolation source: fish sauce Collection date: before 2000 Location: Vietnam	✓	[29]
Bacillus wiedmannii FSL W8-0169	CIP 111364, DSM 102050, LMG 29269	Isolation source: raw milk stored in a dairy powder processing plant silo Collection date: 29-APR-2012 Location: New York state, USA	✓	[30]
Celeribacter baekdonensis L-6	CCUG 60799, DSM 27375, KCTC 23497	Isolation source: seawater Collection date: 2007 Location: Baekdo harbour, East Sea, Korea	✓	[31]
Defluviimonas alba cai42	CGMCC 1.12518, LMG 27406	Isolation source: oil-production water Collection date: JUN-2011 Location: Xinjiang, China	✓	[32]
Gemmobacter tilapiae Ruye-53	BCRC 80261, KCTC 23310	Isolation source: freshwater pond, used to rear tilapiine cichlid fish (*Tilapia rendalli*) Collection date: before 2013 Location: Taitung city, eastern Taiwan (22° 56′ 42″ N / 121° 09′ 18″ E)	✓	[33]
Haematobacter massiliensis CCUG 47968	CIP 107725, framboise	Isolation source: nose of a patient with aspiration pneumonia Collection date: 2003 Location: Marseilles, France	✓	[34]
Paracoccus zeaxanthinifaciens ATCC 21588	CIP 108019, IAM 15326, JCM 21774, LMG 21293, R-1512	Isolation source: seaweed Collection date: before 1975 Location: coast of the African Red Sea	✓	[35]
Pararhodobacter sp. CCB-MM2	N/A	Isolation source: mangrove soil, 10 cm depth Collection date: 02-OCT-2014 Location: Matang Mangrove Forest, Malaysia (4.21335 N / 100.64708999999993 E)	-	[36]
Pararhodobacter sp. CIC4N-9	KCTC 52336, MCCC 1A01225	Isolation source: deep-sea water, 2391 m depth Collection date: 2015-2016 Location: Southwest Indian Ridge, Indian ocean (51.06° E / 37.53° S)	-	[37]
Rhodobacter aestuarii JA296	CCUG 55130, DSM 19945, JCM 14887	Isolation source: mud from brown-coloured microbial mat from brackish water Collection date: 15-JUN-2007 Location: Bhitarkanika mangrove forest, Dangmal, Orissa, India (2° 49′ N 8° 47′ E)	✓	[38]

Rhodobacter sp. Bo10-19	N/A	Isolation source: *Oscillatoria brevis* (filamentous cyanobacteria) culture<br>Collection date: between 1992 and 1994<br>Location: sediment of shallow coastal waters of the Baltic Sea, Boiensdorf, Germany	-	[39]
Rhodobacter sp. LW4	N/A	Isolation source: water<br>Collection date: 25-AUG-2008<br>Location: Lonar Lake, Lonar, Buldhana district, Maharashtra, India	-	FM956479
Rhodobacter sp. R18	N/A	Isolation source: *Nannochloropsis oculata* (phytoplankton)<br>Collection date: before 2011<br>Location: Susami Fish Nursery Center, Kinki University, Japan	-	[40]
Rhodobacter sphaeroides 2.4.1	ATCC 11167, ATCC 14690, ATCC 17023, CCUG 31486, CIP 60.6, DSM 158, IAM 14237, LMG 2827, NCIB 8253	Isolation source: N/A<br>Collection date: before 1944<br>Location: N/A	✓	[41]
Rhodobacter vinaykumarii JA123	DSM 18714, CCUG 54311, CIP 109721, JCM 14544,	Isolation source: tidal seawater<br>Collection date: 16-APR-2005<br>Location: Bay of Bengal, Visakhapatnam, Ramakrishna beach, India	✓	[42]
Roseicitreum antarcticum ZS2-28	CGMCC 1.8894, LMG 24863	Isolation source: coastal region, sandy intertidal sediment<br>Collection date: MAR-2007<br>Location: Chinese Antarctic Zhongshan Station, Larsemann Hills, Princess Elizabeth Land, East Antarctica	✓	[43]
Vibrio alginolyticus ATCC 33787	N/A	Isolation source: seawater<br>Collection date: 05-APR-1971<br>Location: Oahu, Hawaii, USA, (20.3 N 157.3 W)	-	[44]
Vibrio alginolyticus NBRC 15630	ATCC 17749, Baumann 118, CAIM 516, CCM 2578, CIP 75.3, CIP 103336, CCUG 4989, CCUG 13445, CCUG 16315, DSM 2171, IFO 15630, NBRC 15630, NCCB 71013, NCCB 77003, NCTC 12160, NCIMB 1903, LMG 4409, XII-53	Isolation source: spoiled horse mackerel, causing food poisoning<br>Collection date: before 1961<br>Location: Japan	✓	[45]
Vibrio atypicus HHS02	CGMCC 1.8461, DSM 25292, LMG 24781	Isolation source: Digestive tract of healthy Chinese prawns *Penaeus chinensis* O'sbeck<br>Collection date: before 2010<br>Location: Qingdao, China	✓	[46]
Vibrio campbellii CAIM 519	ATCC 25920, Baumann 40, CCM 2582, CCUG 4979, CCRC 12909, CECT 523, CIP 75.01, DSM 19270, Lee MV2189, NCTC 11317, IFO 15631, LMD 73.2, LMG 11216, NBRC 15631, NCIMB 1894, Verdonck VIB 285	Isolation source: seawater, 800 m depth<br>Collection date: before 1971<br>Location: Hawaii, USA (20.5000°/ 157.5000°)	✓	[44]

Vibrio cholerae CECT 514	ATCC 14035, CCUG 9118, CDC 9060-79, CIP 62.13, DSM 100200, JCM 852, KCTC 23507, NCCB 80091, NCCB 36033, NCTC 8021	Isolation source: human fecal sample<br>Collection date: before 1854<br>Location: N/A	✓	[47]
Vibrio diabolicus FDAARGOS_105	ATCC 33810, Baumann 611, Lee 5299, LMG 11654, Shehabi 36, Verdonck VIB 383	Isolation source: human (vibrio infected) feces<br>Collection date: before 1981<br>Location: Amman, Jordan	-	[48]
Vibrio furnissii ATCC 35016	CCUG 37301, CDC B3215, CIP 102972, DSM 19622, FDAARGOS_777, KCTC 2731, LMG 7910, NCTC 13120	Isolation source: human feces of an adult woman with gastroenteritis<br>Collection date: 1969<br>Location: Japan	✓	[49]
Vibrio harveyi FDAARGOS_107	ATCC 43516, LMG 11755, Verdonck VIB 351	Isolation source: mouth of a shark<br>Collection date: before 1985<br>Location: Bahamas	-	[50]
Vibrio natriegens NBRC 15636	ATCC 14048, Baumann 111, DSM 759, CCM 2575, CCUG 16371, CIP 103193, CIP 75.07, NCIMB 857	Isolation source: salt marsh mud<br>Collection date: before 1958<br>Location: Sapelo Island, Georgia, USA	✓	[51]
Vibrio parahaemolyticus ATCC 17802	ATCC 17802, Baumann 113, CAIM 320, CCUG 14474, CCUG 15657, CCTM 2011, CECT 511, CDC 9062-79, CIP 75.02, DSM 10027, EB 101, FDAARGOS_115, FIRDI 806, Lee MV1424, NCIMB 1902, NCTC 10903, RIMD 2210001, USCC 2240, Verdonck VIB 304, WDCM 00037	Isolation source: shirasu (food poisoned victim)<br>Collection date: 1951<br>Location: Japan	✓	[52]
Vibrio proteolyticus NBRC 13287	ATCC 15338, CAIM 511, CCUG 20302, CCEB 710, CIP 710, CIP 73.06, CIP 102892, DSM 30189, IFO 13287, NCIMB 1326, LMG 3772, KCTC 2730, Popoff 262	Isolation source: intestine of *Limnoria tripunctata* (wood boring isopod) from wooden pilings<br>Collection date: before 1958<br>Location: Fort Johnson Marine Laboratory, Charleston, SC, USA	✓	[53]
Vibrio rotiferianus B64D1	N/A	Isolation source: bottom water (17.5 m)<br>Collection date: AUG-2015<br>Location: Bohai Sea, China (38.23 N, 119.04 E)	-	[54]
Vibrio tubiashii ATCC 19109	CAIM 313, CCUG 19118, CCUG 38428, CECT 4196, CIP 102760, DSM 19142, IFO 15644, LMG 10936, NBRC 15644, NCIMB 1340, Milford 74	Isolation source: larvae from hard clams *Mercenaria mercenaria*)<br>Collection date: 1965<br>Location: Long Island Sound, Connecticut, USA	✓	[55]
Vibrio xiamenensis G21	CGMCC 1.10228, DSM 22851	Isolation source: soil beneath mangrove<br>Collection date: before 2010<br>Location: Xiamen, Fujian province, China	✓	[56]

Table S2. 16S rRNA gene sequence information for the taxa included in phylogenetic tree reconstruction.

Species	GenBank accession number	Nucleotide region	Reference
Aliivibrio fischeri ATCC 7744	X74702	1 - 1467	[57]
Anaerobacillus macyae DSM 16346	LELK01000003	66 - 1619	[58]
Bacillus algicola KMM 3737	AY228462	1 - 1545	[3]
Bacillus altitudinis 41KF2b	AJ831842	1 - 1506	[4]
Bacillus amyloliquefaciens DSM 7	FN597644	91526 - 93063	[59]
Bacillus aquimaris TF-12	AF483625	1 - 1507	[6]
Bacillus atrophaeus 1942	CP002207	3638254 - 3639812	[7]
Bacillus atrophaeus JCM9070	AB021181	1 - 1515	[60]
Bacillus cereus ATCC 14579	AE016877	9188 - 10699	[61]
Bacillus cytotoxicus NVH 391-98	AM747234	1 - 1532	[62]
Bacillus firmus EN 5C	KY399766	1 - 1522	[11]
Bacillus firmus NCTC 10335	UFTC01000001	102662 - 104217	Direct submission (06-JUN-2018)
Bacillus gottheilii WCC 4585	FN995266	1 - 1512	[13]
Bacillus hwajinpoensis 22506_14_FS	WMEY01000019	1 - 1466	Direct Submission (07-NOV-2019)
Bacillus hwajinpoensis Y2	SWFM01000018	86 - 1640	Direct Submission (24-APR-2019)
Bacillus infantis NRRL B-14911	CP006643	267863 - 269416	[63]
Bacillus krulwichiae AM31D	AB086897	1 - 1507	[15]
Bacillus marisflavi 151-25	CP047095	364983 - 366536	[16]
Bacillus marisflavi TF-11	AF483624	1 - 1506	[6]
Bacillus megaterium IAM 13418	D16273	1 - 1486	[64]
Bacillus oceanisediminis 2691	CP015506	172748 - 174302	[18]
Bacillus oceanisediminis H2	GQ292772	1 - 1393	[19]
Bacillus oryzaecorticis IHB B 17121	KY933462	1 - 1524	Direct Submission (13-APR-2017)
Bacillus pseudofirmus DSM 8715	X76439	1 - 1506	[65]
Bacillus shackletonii LMG 18435	AJ250318	1 - 1503	[21]
Bacillus sp. BW3PhG2	KC012849	1 - 1545	[22]
Bacillus sp. JL-29	AY646165	1 - 1516	[23]
Bacillus sp. MN-003	AF355627	1 - 1514	[24]
Bacillus sp. N1-1	CP046564	9504 - 11061	[25]
Bacillus subtilis DSM10	AJ276351	1 - 1517	Direct submission (07-MAR-2000)
Bacillus thuringiensis ATCC 10792	CP021061	88791 - 90346	[66]
Bacillus toyonensis BCT-7112	CP006863	2650827 - 2652381	[28]
Bacillus vietnamensis 151-6	CP047394	38967 - 40522	[16]
Bacillus vietnamensis NBRC 101237	BCVQ01000102	79 - 1636	Direct Submission (26-FEB-2016)
Bacillus wiedmannii FSL W8-0169	KU198626	1 - 1540	[30]
Celeribacter baekdonensis L-6	HM997022	1 - 1423	[31]
Defluviimonas alba cai42	CP012661	4102517 - 4103969	[67]
Gemmobacter tilapiae Ruye-53	HQ111526	1 - 1402	[33]
Haematobacter massiliensis CCUG 47968	AF452106	1 - 1414	[34]

Paracoccus zeaxanthinifaciens ATCC 21588	AF461158	1 - 1404	[35]
Pararhodobacter sp. CCB-MM2	LRRR01000059	250 - 1695	[36]
Pararhodobacter sp. CIC4N-9	QEYD01000020	242 - 1709	[37]
Rhodobacter aestuarii JA296	AM748926	1 - 1369	[38]
Rhodobacter sp. Bo10-19	EU839358	1 - 1431	[39]
Rhodobacter sp. LW4	FM956479	1 - 1445	Direct submission (09-JAN-2009)
Rhodobacter sp. R18	AB607872	1 - 1430	[40]
Rhodobacter sphaeroides 2.4.1	CP030271	1 - 1471	[68]
Rhodobacter vinaykumarii JA123	AM408117	1 - 1387	[42]
Roseicitreum antarcticum ZS2-28	FJ196006	1 - 1429	[43]
Vibrio alginolyticus ATCC 33787	CP013484	2195014 - 2196575	[69]
Vibrio alginolyticus NBRC 15630	CP006718	1961445 - 1962985	[70]
Vibrio atypicus HHS02	FJ009624	1 - 1401	[46]
Vibrio campbellii CAIM 519	CP015863	649525 - 651086	Direct Submission (25-MAY-2016)
Vibrio cholerae CECT 514	X76337	1 - 1538	[71]
Vibrio diabolicus FDAARGOS_105	CP014036	427222 - 428783	[72]
Vibrio furnissii ATCC 35016	X76336	1 - 1546	[71]
Vibrio harveyi FDAARGOS_107	CP014038	1618778 - 1620338	[72]
Vibrio natriegens NBRC 15636	CP016345	2855557 - 2857118	[73]
Vibrio parahaemolyticus ATCC 17802	CP014046	900558 - 902118	[72]
Vibrio proteolyticus NBRC 13287	BATJ01000038	1 - 1562	Direct Submission (10-SEP-2013)
Vibrio rotiferianus B64D1	CP018312	4099 - 5660	[54]
Vibrio tubiashii ATCC 19109	CP009354	47507 - 49071	[74]
Vibrio xiamenensis G21	GQ397859	1 - 1475	[56]

Table S3. Genome availability of species in NCBI used for phylogenetic analysis and comparative genomics. Included are the GenBank accession number, assembly level, and representative genome status for a given species.

Species	GenBank accession number	Assembly level	Ref. seq.	Reference
Aliivibrio fischeri ATCC 7744	BBEE00000000 180 contigs: BBEE01000001-BBEE01000180	◔ Contigs	-	Direct submission (18-JUN-2014)
Anaerobacillus macyae DSM 16346	LELK00000000 15 scaffolds: LELK01000001-LELK01000015	◐ Scaffolds	✓	[58]
Bacillus algicola KMM 3737	No genome	○ No genome	-	N/A
Bacillus altitudinis 41KF2b	ASJC00000000 39 contigs: ASJC01000001-ASJC01000039	◔ Contigs	-	Direct submission (09-MAY-2013)
Bacillus amyloliquefaciens DSM 7	Chr: FN597644	● Complete	✓	[59]
Bacillus aquimaris TF-12	LQXM00000000 30 contigs: LQXM01000001-LQXM01000030	◔ Contigs	✓	[75]
Bacillus atrophaeus 1942	Chr: CP002207	● Complete	-	[7]
Bacillus atrophaeus JCM9070	LSBB00000000 23 scaffolds: LSBB01000001-LSBB01000023	◐ Scaffolds	-	[76]
Bacillus cereus ATCC 14579	Chr: CP034551; pUnnamed: CP034552	● Complete	✓	Direct submission (13-DEC-2018)
Bacillus cytotoxicus NVH 391-98	Chr: CP000764; pBC9801: CP000765	● Complete	✓	[77]
Bacillus firmus EN 5C	No genome	○ No genome	-	N/A
Bacillus firmus NCTC 10335	UFTC00000000 3 contigs: UFTC01000001-UFTC01000003	◔ Contigs	✓	Direct submission (06-JUN-2018)
Bacillus gottheilii WCC 4585	No genome	○ No genome	-	N/A
Bacillus hwajinpoensis 22506_14_FS	WMEY00000000 27 contigs: WMEY01000001-WMEY01000027	◔ Contigs	-	Direct submission (07-NOV-2019)
Bacillus hwajinpoensis Y2	SWFM00000000 25 contigs: SWFM01000001-SWFM01000025	◔ Contigs	✓	Direct submission (24-APR-2019)
Bacillus infantis NRRL B-14911	Chr: CP006643	● Complete	✓	[63]
Bacillus krulwichiae AM31D	Chr: CP020814	● Complete	✓	Direct submission (18-APR-2017)
Bacillus marisflavi TF-11	LGUE00000000 11 scaffolds: LGUE01000001-LGUE01000011	◐ Scaffolds	✓	[78]
Bacillus marisflavi 151-25	Chr: CP047095; p25: CP047096	● Complete	-	[16]
Bacillus megaterium IAM 13418	Chr: CP009920; pBMV_1: CP009919; pBMV_2: CP009921; pBMV_3: CP009915; pBMV_4: CP009918; pBMV_5: CP009916; pBMV_6: CP009917	● Complete	✓	[79]
Bacillus oceanisediminis H2	VLKI00000000 51 scaffolds: VLKI01000001-VLKI01000051	◐ Scaffolds	-	[80]

Bacillus oceanisediminis 2691	Chr: CP015506<br>pBO1: CP015507	● Complete	✓	[18]
Bacillus oryzaecorticis IHB B 17121	No genome	○ No genome	-	N/A
Bacillus pseudofirmus DSM 8715	No genome	○ No genome	-	N/A
Bacillus shackletonii LMG 18435	LJJC00000000.1<br>24 scaffolds: LJJC01000001-LJJC01000024	◐ Scaffolds	✓	[81]
Bacillus sp. BW3PhG2	Chr: CP046564	● Complete	-	[25]
Bacillus sp. JL-29	No genome	○ No genome	-	N/A
Bacillus sp. MN-003	No genome	○ No genome	-	N/A
Bacillus sp. N1-1	No genome	○ No genome	-	N/A
Bacillus subtilis DSM10	Chr: CP020102;<br>pBS32: CP020103	● Complete	-	[82]
Bacillus thuringiensis ATCC 10792	Chr: CP021061; poh1: CP021062;<br>poh2: CP021063; poh3: CP021064;<br>poh4: CP021065; poh5: CP021066	● Complete	✓	[66]
Bacillus toyonensis BCT-7112	Chr: CP006863; pBCT77: CP006864;<br>pBCT8: CP006865	● Complete	✓	[28]
Bacillus vietnamensis 151-6	Chr: CP047394<br>p6: CP047395	● Complete	-	[16]
Bacillus vietnamensis NBRC 101237	BCVQ00000000<br>123 contigs: BCVQ01000001-BCVQ01000123	◔ Contigs	✓	Direct submission (26-FEB-2016)
Bacillus wiedmannii FSL W8-0169	LOBC00000000<br>104 contigs: LOBC01000001-LOBC01000104	◔ Contigs	-	[30]
Celeribacter baekdonensis L-6	FNBL00000000<br>38 scaffolds: FNBL01000001-FNBL01000038	◐ Scaffolds	✓	Direct submission (11-OCT-2016)
Defluviimonas alba cai42	Chr: CP012661; cai42_plasmidA: CP012662;<br>cai42_plasmidB: CP012663;<br>cai42_plasmidC: CP012664	● Complete	✓	[67]
Gemmobacter tilapiae Ruye-53	No genome	○ No genome	-	N/A
Haematobacter massiliensis CCUG 47968	JGYG00000000<br>53 contigs: JGYG01000001-JGYG01000053	◔ Contigs	-	Direct submission (04-MAR-2014)
Paracoccus zeaxanthinifaciens ATCC 21588	ATUJ00000000<br>35 contigs: ATUJ01000001-ATUJ01000035	◔ Contigs	✓	Direct submission (02-JUL-2013)
Pararhodobacter sp. CIC4N-9	QEYD00000000<br>30 contigs: QEYD01000001-QEYD01000030	◔ Contigs	-	[37]
Pararhodobacter sp. CCB-MM2	LRRR00000000<br>136 contigs: LRRR01000001-LRRR01000136	◔ Contigs	-	[36]
Rhodobacter aestuarii JA296	FTOG00000000<br>31 contigs: NZ_FTOG01000001-NZ_FTOG01000031	◔ Contigs	✓	Direct submission (09-JAN-2017)
Rhodobacter sp. Bo10-19	No genome	○ No genome	-	N/A
Rhodobacter sp. LW4	No genome	○ No genome	-	N/A

Rhodobacter sp.<br>R18	No genome	○<br>No genome	-	N/A
Rhodobacter sphaeroides<br>2.4.1	Chr1: CP030271; Chr2: CP030272;<br>pA: CP030273; pB: CP030274;<br>pC: CP030275; pDE: CP030276	●<br>Complete	✓	[68]
Rhodobacter vinaykumarii<br>JA123	FTOM00000000<br>27 scaffolds: FTOM01000001-FTOM01000027	◐<br>Scaffolds	✓	Direct submission<br>(09-JAN-2017)
Roseicitreum antarcticum<br>ZS2-28	FNOM00000000<br>77 scaffolds: FNOM01000001-<br>FNOM01000077	◐<br>Scaffolds	✓	Direct submission<br>(20-OCT-2016)
Vibrio alginolyticus<br>ATCC 33787	Chr1: CP013484; Chr2: CP013485;<br>pMBL96: CP013488; pMBL128: CP013486;<br>pMBL287: CP013487	●<br>Complete	-	[69]
Vibrio alginolyticus<br>NBRC 15630	Chr1: CP006718;<br>Chr2: CP006719	●<br>Complete	✓	[70]
Vibrio atypicus<br>HHS02	BLID00000000<br>66 scaffolds: BLID01000001-BLID01000066	◐<br>Scaffolds	✓	[83]
Vibrio campbellii<br>CAIM 519	Chr1: CP015863; Chr2: CP015864;<br>pUnnamend: CP015865	●<br>Complete	-	Direct submission<br>(25-MAY-2016)
Vibrio cholerae<br>CECT 514	JHXR00000000<br>62 scaffolds: JHXR01000001-JHXR01000004;<br>JHXR01000009-JHXR01000011;<br>JHXR01000014-JHXR01000024;<br>JHXR01000027-JHXR01000034;<br>JHXR01000038-JHXR01000068; KK211322-<br>KK211326	◐<br>Scaffolds	-	Direct submission<br>(26-MAR-2014)
Vibrio diabolicus<br>FDAARGOS_105	Chr1: CP014036;<br>Chr2: CP014037	●<br>Complete	✓	[72]
Vibrio furnissii<br>ATCC 35016	Chr1: CP040990; Chr2: CP040991;<br>pUnnamed1: CP040989	●<br>Complete	✓	[72]
Vibrio harveyi<br>FDAARGOS_107	Chr1: CP014038.2;<br>Chr2: CP014039.2	●<br>Complete	✓	[72]
Vibrio natriegens<br>NBRC 15636	Chr1: CP016345;<br>Chr2: CP016346	●<br>Complete	-	[73]
Vibrio parahaemolyticus<br>ATCC 17802	Chr1: CP014046;<br>Chr2: CP014047	●<br>Complete	✓	[72]
Vibrio proteolyticus<br>NBRC 13287	BATJ01000000<br>50 contigs: BATJ01000001-BATJ01000050	◔<br>Contigs	✓	Direct submission<br>(10-SEP-2013)
Vibrio rotiferianus<br>B64D1	Chr1: CP018311;<br>Chr2: CP018312	●<br>Complete	✓	[54]
Vibrio tubiashii<br>ATCC 19109	Chr1: CP009354; Chr2: CP009355;<br>p48: CP009359; p57: CP009358<br>p123: CP009357; p251: CP009356	●<br>Complete	✓	[74]
Vibrio xiamenensis<br>G21	FNDD00000000<br>82 scaffolds: FNDD01000001-FNDD01000082	◐<br>Scaffolds	✓	Direct submission<br>(12-OCT-2016)

Table S4. Properties of the genomes used for the *Bacillaceae* comparative genomics analysis with Anvi'o. NTK strains are marked with a (◀).

Species	Number gene clusters	Singleton gene clusters	Completion (%)	GC-content	Total length (bp)
Bacillus sp. NTK034 ◀	5617	838	100	0.409657	5599963
Bacillus oceanisediminis 2691	5850	1089	100	0.408836	5848607
Bacillus oceanisediminis H2	5590	1343	100	0.408821	5634660
Bacillus infantis NRRL B-14911	5031	1622	98.59	0.460333	4884713
Bacillus firmus NCTC 10335	4541	599	100	0.417216	4803910
Bacillus vietnamensis NBRC 101237	4538	366	100	0.437364	4410879
Bacillus vietnamensis 151-6	4694	542	100	0.436474	4597807
Bacillus sp. NTK074B ◀	4668	846	100	0.435474	4250699
Bacillus aquimaris TF-12	3862	690	100	0.373054	4035445
Bacillus marisflavi TF-11	4390	1170	100	0.485657	4312088
Bacillus sp. NTK071 ◀	4136	344	100	0.398513	4164462
Bacillus hwajinpoensis Y2	4020	242	100	0.397388	4091520
Anaerobacillus macyae DSM 16346	4149	315	100	0.398453	4256776
Bacillus sp. N1-1	4389	332	100	0.404822	4497340
Bacillus hwajinpoensis 22506_14_FS	4415	355	100	0.402183	4362982

Table S5. Pangenomic analysis properties of the *Rhodobacteraceae* genomes that were compared with NTK community NTK016B (◀).

Species	Number gene clusters	Singleton gene clusters	Completion (%)	GC-content	Total length (bp)
Rhodobacter sphaeroides 2.4.1	4361	2074	100	0.687717	4629754
Defluviimonas alba cai42	4770	2257	100	0.665331	4987611
Roseicitreum antarcticum ZS2-28	4046	1845	100	0.631032	4250016
Rhodobacter sp. NTK016B ◀	4644	790	100	0.653535	4854159
Pararhodobacter sp. CIC4N-9	4299	499	100	0.667462	4486134
Pararhodobacter sp. CCB-MM2	4813	1431	100	0.659524	5149122

Table S6. Properties of genomes used for the *Vibrionaceae* pangenomic analysis with the NTK community strains (◀).

Species	Number gene clusters	Singleton gene clusters	Completion (%)	GC-content	Total length (bp)
Vibrio furnissii ATCC 35016	4544	1044	100	0.505984	4993326
Vibrio proteolyticus NBRC 13287 ◀	4342	747	100	0.500146	4745539
Vibrio tubiashii ATCC 19109	4979	1210	100	0.450052	5540337
Vibrio atypicus HHS02	4303	685	100	0.438952	4835723
Vibrio parahaemolyticus ATCC 17802	4557	340	100	0.45330	5152461
Vibrio diabolicus FDAARGOS_105	4834	489	100	0.44815	5426154
Vibrio alginolyticus NBRC 15630	4506	142	100	0.446793	5146637
Vibrio alginolyticus ATCC 33787 ◀	5174	778	100	0.445122	5724939
Vibrio natriegens NBRC 15636	4500	811	100	0.450796	5168686
Vibrio rotiferianus B64D1	4602	443	100	0.448405	5277859
Vibrio harveyi FDAARGOS_107	5332	745	100	0.449027	6038929
Vibrio campbellii CAIM 519	4667	624	100	0.450805	5178103

Table S7. Amino acid sequences of the extracellular PHA depolymerase candidates.

Species	Annotated protein name	Amino acid sequence
Bacillus sp. NTK074B	Hypothetical protein	MIKKISMTVLALFLLLPSWSHAGSWTKDPSGVPGQWYVGTTPSSVAPSKQPILFVH GLNSSSNTWWNDNNMYDTAYQNGYETAFIDLYPTRNMWDNGTLLSQKIRDIYN YYGEKVVIVAHSKGGIDVQSALVHYGAYPYVSRVITLSTPHYGSQLADLAYSSWAG WLAGILGSKNEATYSLQTGYMSYFREETDQNANVTKVPFYTFGGTKWGSFGSSLY WGGLYLSGYGSNDGAVTVNSSRLPYATELKVGGWNHYTIKEGSSTFNLFKGYLNE TTNNSSGAVTQSASFNNDADSFFRGGGYSGETQEKILVEEGARSVTFDWMSSSPSSR LVLKDPKKNKYSSFDVSKDNSDYFNGAYHHTLTINTPNPGEWTLESASQSTETYLL NVSFDSIMNNAVSMSMEQDQISLKKKDSDVSIQQDMTIEYYKNGKLKESKLKSKN GLLKLPSLGEGVYNVTIDIKGKKGKNAFNRTLIKTIYVDDKGRIFGE
Vibrio alginolyticus ATCC 33787	Lipase precursor (EC 3.1.1.3)	MKLKFIALMLFTLVTVPITSVSAATGYTQTKYPIVLVHGLFGFDTLAGVDYFFGIPHS LTKDGATVYVAQVSATNSSEVRGEQLLAQVETLLAATGAEKVNLIGHSHGGPTTR YVASVRPDLVASVTSIGGVNKGSKVADLVRGTVSEGSGSEQLAVKLAQGLTTLINL LSGGSDLDQDPLASLATLTTEGSLAFNQHYPEGVPTSECGNGDLLASNGVYYYSWT GSSTFTNVFDPTDAAMMVLGLAFDGPNDGLVGACSTHLGKVIRDDYQMNHLDEI NGLLGIHHLFETDPVTLYRQHANRLKLQGL
Vibrio proteolyticus NBRC 13287	Lipase precursor (EC 3.1.1.3)	MKLYLVALCLFSPFVLASTSASALDNTGYTQTRYPIVLVHGLFGFDTLAGVDYFYSI PHSLTKDGASVYVAQVSATNSSELRGEQLLSQVEMLLAATGAEKVNLIGHSHGGP TARYVASVRPDLVASVTSVGGVNKGSKVADLVRGAVADGSAGEAIAVKLAEGLV TLINLLSGGSDLEQDPLASLAALTTEGSLAFNQHYPEGVPTSECGDGEFLADNGVY YYSWTGSSTFTNLLDPTDAAMTILGLAFDGPSDGLVGVCSTHLGKVIRDDYQMNH LDEINGLLGVHHLFETDPVTLYRQHANRLKLQGL

Table S8. Positions of conserved sites in known extracellular PHA depolymerases and closely related lipases, with candidate extracellular PhaZs (◀). Proteins with experimentally validated positions are marked bold. If no experimental evidence was published on the proteins, signal peptides were predicted by SignalP and conserved sites were aligned visually with the validated proteins.

Protein	NCBI Protein Accession	Species	Signal peptide	Oxyanion hole	Lipase box	Catalytic triad (S-H-A)	Reference
Hypothetical protein ◀	XXXXXX	*Bacillus* sp. NTK074B	1-22	57-57	119-123	121-223-256	This study
Hypothetical protein sed_3530	ABV38134	*Shewanella sediminis* HAW-EB3	1-23	67-73	160-164	162-269-331	[84]
**Lipase A**	**P37957**	***Bacillus subtilis* strain 168**	**1-31**	**36-42**	**106-110**	**108-164-187**	**[85]**
Lipase class 2	ABZ77296	*Shewanella halifaxensis* HAW-EB4	1-23	67-73	160-164	162-269-331	[84]
Lipase precursor ◀	XXXXXX	*Vibrio alginolyticus* ATCC 33787	1-23	33-39	104-108	106-253-275	This study
Lipase precursor ◀	XXXXXX	*Vibrio proteolyticus* NBRC 13287	1-23	35-41	106-110	108-255-277	This study
**PhaZ7**	**Q939Q9**	***Paucimonas lemoignei***	**1-38**	**80-86**	**172-176**	**174-280-344**	**[86-88]**
**Thermostable lipase**	**Q842J9**	***Geobacillus zalihae* T1**	**1-28**	**37-43**	**139-143**	**141-345-386**	**[89,90]**
Triacylglycerol lipase	WP_005101273	*Acinetobacter* multispecies	1-29	50-56	125-129	127-280-302	[91]
Triacylglycerol lipase	WP_005101276	*Acinetobacter* multispecies	1-23	44-50	120-124	122-267-289	[91]
Triacylglycerol lipase	WP_064094572	*Acinetobacter* multispecies	1 - 21	42-48	117-121	119-266-288	[91]
**Triacylglycerol lipase**	**P22088**	***Burkholderia cepacia* ATCC 21808**	**1-44**	**54-60**	**129-133**	**131-308-330**	**[92,93]**
**Triacylglycerol lipase**	**Q05489**	***Burkholderia glumae* ATCC 6918**	**1-39**	**49-55**	**124-128**	**126-302-324**	**[94,95]**
**Triacylglycerol lipase**	**P26876**	***Pseudomonas aeruginosa* PAO1**	**1-26**	**35-41**	**106-110**	**108-255-277**	**[96,97]**

Table S9. MiXS table with metadata for sequenced NTK isolate rRNA marker genes (MIMARKS).

Structured comment name	***Bacillus* sp. NTK_Randy**	***Bacillus* sp. NTK029**	***Bacillus* sp. NTK072**
investigation_type	mimarks-specimen	mimarks-specimen	mimarks-specimen
project_name	NTK biodegradation consortium	NTK biodegradation consortium	NTK biodegradation consortium
experimental_factor	N/A	N/A	Polycaprolactone surface incubated for 8 weeks in seawater
lat_lon	42.652730 -70.688200	N/A	N/A
geo_loc_name	USA;Massachusetts;Wingaersheek Beach (Gloucester)	Pacific Ocean;Hawaii (USA)	Atlantic Ocean;United States northeast coast
collection_date	N/A	N/A	N/A
env_broad_scale	Marine benthic biome [ENVO:01000024]	Ocean biome [ENVO:01000048]	Ocean biome [ENVO:01000048]
env_local_scale	Coastal zone [ENVO:00000486]	Ocean water environment [ENVO:01000321]	Coastal zone [ENVO:00000486]
env_medium	Coastal ocean water [ENVO:00002150]	Ocean water [ENVO:00002149]	Coastal ocean water [ENVO:00002150]
isol_growth_condt	doi: T.B.D	doi: T.B.D	doi: T.B.D
target_gene	16S rRNA	16S rRNA	16S rRNA
pcr_primers	FWD:GTTTGATCCTGGCTCAG;REV: TACCTTGTTACGACTT	FWD:GTTTGATCCTGGCTCAG;REV:TACCTTGTTACGACTT	FWD:GTTTGATCCTGGCTCAG;REV:TACCTTGTTACGACTT
seq_meth	Sanger	Sanger	Sanger
seq_quality_check	manually edited	manually edited	manually edited

Table S10. MiXS tables with metadata for sequenced NTK isolate genomes (MIGS).

Section	Item	Structured comment name	*Rhodobacter* sp. NTK016B	*Bacillus* sp. NTK034	*Bacillus* sp. NTK071	*Bacillus* sp. NTK074B
investigation	investigation type	investigation_type	bacteria_archaea	bacteria_archaea	bacteria_archaea	bacteria_archaea
investigation	project name	project_name	NTK biodegradation consortium	NTK biodegradation consortium	NTK biodegradation consortium	NTK biodegradation consortium
investigation	experimental factor	experimental_factor	Polycaprolactone biodegradation experiment	N/A	Polycaprolactone surface incubated for 8 weeks in seawater	Polycaprolactone surface incubated for 8 weeks in seawater
environment	geographic location (latitude and longitude)	lat_lon	N/A	N/A	N/A	N/A
environment	geographic location (country and/or sea,region)	geo_loc_name	Atlantic Ocean;United States northeast coast	Pacific Ocean;Hawaii (USA)	Atlantic Ocean;United States northeast coast	Atlantic Ocean;United States northeast coast
environment	collection date	collection_date	N/A	N/A	N/A	N/A
environment	broad-scale environmental context	env_broad_scale	Marine benthic biome [ENVO:01000024]	Ocean biome [ENVO:01000048]	Ocean biome [ENVO:01000048]	Ocean biome [ENVO:01000048]
environment	local environmental context	env_local_scale	Coastal zone [ENVO:00000486]	Ocean water environment [ENVO:01000321]	Coastal zone [ENVO:00000486]	Coastal zone [ENVO:00000486]
environment	environmental medium	env_medium	Marine sediment [ENVO:03000033]	Ocean water [ENVO:00002149]	Coastal ocean water [ENVO:00002150]	Coastal ocean water [ENVO:00002150]
nucleic acid sequence source	number of replicons	num_replicons	N/A	N/A	N/A	N/A
nucleic acid sequence source	reference for biomaterial	ref_biomaterial	doi: T.B.D.	doi: T.B.D.	doi: T.B.D.	doi: T.B.D.
nucleic acid sequence source	isolation and growth condition	isol_growth_condt	doi: T.B.D.	doi: T.B.D.	doi: T.B.D.	doi: T.B.D.
sequencing	library layout	lib_layout	paired	paired	paired	paired
sequencing	adapters	adapters	CGCTACAT;CGCTACAT	AATCCAGC;AATCCAGC	CGTCTAAC;CGTCTAAC	AACTCGGA;AACTCGGA
sequencing	sequencing method	seq_meth	Illumina NextSeq 500	Illumina NextSeq 500	Illumina NextSeq 500	Illumina NextSeq 500
sequencing	taxonomic identity marker	tax_ident	16S rRNA gene	16S rRNA gene	16S rRNA gene	16S rRNA gene

sequencing	assembly quality	assembly_qual	High-quality draft genome	High-quality draft genome	High-quality draft genome	High-quality draft genome
sequencing	assembly software	assembly_software	SPAdes;3.11.1;careful, kmer set 21,33,55, default parameters otherwise	SPAdes;3.11.1;careful, kmer set 21,33,55,77,99, default parameters otherwise	SPAdes;3.11.1;careful, kmer set 21,33,55,77,99, default parameters otherwise	SPAdes;3.11.1;careful, kmer set 21,33,55,77, default parameters otherwise
sequencing	annotation	annot	RASTtk	RASTtk	RASTtk	RASTtk
sequencing	number of contigs	number_contig	162	100	229	446
sequencing	completeness score	compl_score	high;100%	high;100%	high;100%	high;100%
sequencing	completeness software	compl_software	anvi'o;6.1	anvi'o;6.1	anvi'o;6.1	anvi'o;6.1

References

1. Robertson, L.A.; Figge, M.J.; Dunlap, P.V. Beijerinck and the bioluminescent bacteria: Microbiological experiments in the late 19th and early 20th centuries. *FEMS Microbiol. Ecol.* **2011**, *75*, 185–194, doi:10.1111/j.1574-6941.2010.01004.x.
2. Santini, J.M.; Stolz, J.F.; Macy, J.M. Isolation of a new arsenate-respiring bacterium—Physiological and phylogenetic Studies. *Geomicrobiol. J.* **2002**, *19*, 41–52, doi:10.1080/014904502317246156.
3. Ivanova, E.P.; Alexeeva, Y.A.; Zhukova, N.V.; Gorshkova, N.M. *Bacillus algicola* sp. nov., a novel filamentous organism isolated from brown alga *Fucus evanescens*. *Syst. Appl. Microbiol.* **2004**, *27*, 301, doi:10.1078/0723-2020-00269.
4. Shivaji, S.; Chaturvedi, P.; Suresh, K. *Bacillus aerius* sp. nov., *Bacillus aerophilus* sp. nov., *Bacillus stratosphericus* sp. nov. and *Bacillus altitudinis* sp. nov., isolated from cryogenic tubes used for collecting air samples from high altitudes. *Int. J. Syst. Evol. Microbiol.* **2006**, *56*, 1465–1473 doi:10.1099/ijs.0.64029-0.
5. Fukumoto, J. Studies on the production of bacterial amylase. I. Isolation of bacteria secreting potent amylases and their distribution. *Nippon Nogeikagaku Kaishi* **1943**, *19*, 487–503.
6. Yoon, J.-H.; Kim, I.-G.; Kang, K.H.; Oh, T.-K.; Park, Y.-H. *Bacillus marisflavi* sp. nov. and *Bacillus aquimaris* sp. nov., isolated from sea water of a tidal flat of the Yellow Sea in Korea. *J. Med. Microbiol.* **2003**, *53*, 1297–1303, doi:10.1099/ijs.0.02365-0.
7. Gibbons, H.S.; Broomall, S.M.; McNew, L.A.; Daligault, H.; Chapman, C.; Bruce, D.; Karavis, M.; Krepps, M.; McGregor, P.A.; Hong, C. Genomic signatures of strain selection and enhancement in *Bacillus atrophaeus* var. *globigii*, a historical biowarfare simulant. *PLoS ONE* **2011**, *6*, e17836, doi:10.1371/journal.pone.0017836.
8. Nakamura, L. Taxonomic relationship of black-pigmented *Bacillus subtilis* strains and a proposal for *Bacillus atrophaeus* sp. nov. *Int. J. Syst. Evol. Microbiol.* **1989**, *39*, 295–300, doi:10.1099/00207713-39-3-295.
9. Frankland, G.C.; Frankland, P.F. XI. Studies on some new micro-organisms obtained from air. *Philos. Trans. R. Soc. Lond.* **1887**, 257–287, doi:10.1098/rstb.1887.0011.
10. Lund, T.; De Buyser, M.L.; Granum, P.E. A new cytotoxin from *Bacillus cereus* that may cause necrotic enteritis. *Mol. Biotechnol.* **2000**, *38*, 254–261, doi:10.1046/j.1365-2958.2000.02147.x.
11. Balakrishnan, S.L.; Rao, P.P. Monocrotophos Degradation Potential of Bacterial Isolates Isolated from Agricultural Soils of Visakhapatnam Dist. *J. Pure Appl. Microbiol.* **2019**, *13*, 393–402, doi:10.22207/JPAM.13.1.43.
12. Werner, W.E.G. *Botanische Beschreibung Häufiger am Buttersäureabbau Beteiligter Sporenbildender Bakterienspezies*; Gustav Fischer Verlag: Jena, Germany, 1933; Volume 87.
13. Seiler, H.; Wenning, M.; Schmidt, V.; Scherer, S. *Bacillus gottheilii* sp. nov., isolated from a pharmaceutical manufacturing site. *Int. J. Syst. Evol. Microbiol.* **2013**, *63*, 867–872, doi:10.1099/ijs.0.036277-0.
14. Siefert, J.L.; Larios-Sanz, M.; Nakamura, L.K.; Slepecky, R.A.; Paul, J.H.; Moore, E.R.; Fox, G.E.; Jurtshuk Jr, P. Phylogeny of marine *Bacillus* isolates from the Gulf of Mexico. *Curr. Microbiol.* **2000**, *41*, 84–88, doi:10.1007/s002840010098.
15. Yumoto, I.; Yamaga, S.; Sogabe, Y.; Nodasaka, Y.; Matsuyama, H.; Nakajima, K.; Suemori, A. *Bacillus krulwichiae* sp. nov., a halotolerant obligate alkaliphile that utilizes benzoate and m-hydroxybenzoate. *J. Med. Microbiol.* **2003**, *53*, 1531–1536, doi:10.1099/ijs.0.02596-0.
16. Yu, X.; Ding, Z.; Ji, Y.; Zhao, J.; Liu, X.; Tian, J.; Wu, N.; Fan, Y. An operon consisting of a P-type ATPase gene and a transcriptional regulator gene responsible for cadmium resistances in *Bacillus vietamensis* 151–6 and *Bacillus marisflavi* 151–25. *BMC Microbiol.* **2020**, *20*, 18, doi:10.1186/s12866-020-1705-2.
17. de Bary, A. *Vergleichende Morphologie und Biologie der Pilze, Mycetozoen und Bacterien*; Wilhelm Engelmann: Leipzig, Germany, 1884.
18. Jung, J.; Jeong, H.; Kim, H.J.; Lee, D.-W.; Lee, S.J. Complete genome sequence of *Bacillus oceanisediminis* 2691, a reservoir of heavy-metal resistance genes. *Mar. Genome* **2016**, *30*, 73–76, doi:10.1016/j.margen.2016.07.002.
19. Zhang, J.; Wang, J.; Fang, C.; Song, F.; Xin, Y.; Qu, L.; Ding, K. *Bacillus oceanisediminis* sp. nov., isolated from marine sediment. *Int. J. Syst. Evol. Microbiol.* **2010**, *60*, 2924–2929, doi:10.1099/ijs.0.019851-0.
20. Nielsen, P.; Fritze, D.; Priest, F.G. Phenetic diversity of alkaliphilic *Bacillus* strains: Proposal for nine new species. *Microbiology* **1995**, *141*, 1745–1761, doi:10.1099/13500872-141-7-1745.
21. Logan, N.; Lebbe, L.; Hoste, B.; Goris, J.; Forsyth, G.; Heyndrickx, M.; Murray, B.; Syme, N.; Wynn-Williams, D.; De Vos, P. Aerobic endospore-forming bacteria from geothermal environments in northern Victoria Land, Antarctica, and Candlemas Island, South Sandwich archipelago, with the proposal of *Bacillus fumarioli* sp. nov. *Int. J. Syst. Evol. Microbiol.* **2000**, *50*, 1741–1753, doi:10.1099/00207713-50-5-1741.
22. Shen, M.; Yang, R.; Luo, Q.; Wang, S.; Ren, J. Microbial diversity of *Pyropia haitanensis* phycosphere during cultivation. *Wei Sheng Wu Xue Bao* **2013**, *53*, 1087–1102.

23. Du, H.; Jiao, N.; Hu, Y.; Zeng, Y. Diversity and distribution of pigmented heterotrophic bacteria in marine environments. *FEMS Microbiol. Ecol.* **2006**, *57*, 92–105, doi:10.1111/j.1574-6941.2006.00090.x.
24. Zhuang, W.-Q.; Tay, J.-H.; Maszenan, A.; Tay, S. *Bacillus naphthovorans* sp. nov. from oil-contaminated tropical marine sediments and its role in naphthalene biodegradation. *Appl. Microbiol. Biotechnol.* **2002**, *58*, 547–554, doi:10.1007/s00253-001-0909-0.
25. Wang, K.; Zhao, Y.; Wang, X.; Qu, C.; Miao, J. Complete genome sequence of *Bacillus* sp. N1-1, a κ-selenocarrageenan degrading bacterium isolated from the cold seep in the South China Sea. *Mar. Genome* **2020**, 100771, doi:10.1016/j.margen.2020.100771.
26. Conn, H.J. The identity of *Bacillus subtilis*. *J. Infect. Dis.* **1930**, *46*, 341–350.
27. Berliner, E. Über die Schlaffsucht der Mehlmottenraupe (*Ephestia kühniella Zell.*) und ihren Erreger *Bacillus thuringiensis* n. sp. *Z. Angew. Entomol.* **1915**, *2*, 29–56, doi:10.1111/j.1439-0418.1915.tb00334.x.
28. Jiménez, G.; Blanch, A.R.; Tamames, J.; Rosselló-Mora, R. Complete genome sequence of *Bacillus toyonensis* BCT-7112^T, the active ingredient of the feed additive preparation Toyocerin. *Genome Announc.* **2013**, *1*, doi:10.1128/genomeA.01080–13.
29. Noguchi, H.; Uchino, M.; Shida, O.; Takano, K.; Nakamura, L.K.; Komagata, K. *Bacillus vietnamensis* sp. nov., a moderately halotolerant, aerobic, endospore-forming bacterium isolated from Vietnamese fish sauce. *Int. J. Syst. Evol. Microbiol.* **2004**, *54*, 2117–2120, doi:10.1099/ijs.0.02895-0.
30. Miller, R.A.; Beno, S.M.; Kent, D.J.; Carroll, L.M.; Martin, N.H.; Boor, K.J.; Kovac, J. *Bacillus wiedmannii* sp. nov., a psychrotolerant and cytotoxic *Bacillus cereus* group species isolated from dairy foods and dairy environments. *Int. J. Syst. Evol. Microbiol.* **2016**, *66*, 4744, doi:10.1099/ijsem.0.001421.
31. Lee, S.-Y.; Park, S.; Oh, T.-K.; Yoon, J.-H. *Celeribacter baekdonensis* sp. nov., isolated from seawater, and emended description of the genus *Celeribacter* Ivanova *et al*. 2010. *Int. J. Syst. Evol. Microbiol.* **2012**, *62*, 1359–1364, doi:10.1099/ijs.0.032227-0.
32. Pan, X.-C.; Geng, S.; Lv, X.-L.; Mei, R.; Jiangyang, J.-H.; Wang, Y.-N.; Xu, L.; Liu, X.-Y.; Tang, Y.-Q.; Wang, G.-J.; et al. *Defluviimonas alba* sp. nov., isolated from an oilfield. *Int. J. Syst. Evol. Microbiol.* **2015**, *65*, 1805–1811, doi:10.1099/ijs.0.000181.
33. Sheu, S.-Y.; Sheu, D.-S.; Sheu, F.-S.; Chen, W.-M. *Gemmobacter tilapiae* sp. nov., a poly-β-hydroxybutyrate-accumulating bacterium isolated from a freshwater pond. *Int. J. Syst. Evol. Microbiol.* **2013**, *63*, 1550–1556, doi:10.1099/ijs.0.044735-0.
34. Greub, G.; Raoult, D. *Rhodobacter massiliensis* sp. nov., a new amoebae-resistant species isolated from the nose of a patient. *Res. Microbiol.* **2003**, *154*, 631–635, doi:10.1016/j.resmic.2003.08.002.
35. Berry, A.; Janssens, D.; Hümbelin, M.; Jore, J.P.; Hoste, B.; Cleenwerck, I.; Vancanneyt, M.; Bretzel, W.; Mayer, A.F.; Lopez-Ulibarri, R. *Paracoccus zeaxanthinifaciens* sp. nov., a zeaxanthin-producing bacterium. *Int. J. Syst. Evol. Microbiol.* **2003**, *53*, 231–238, doi:10.1099/ijs.0.02368-0.
36. Sam, K.-K.; Lau, N.-S.; Furusawa, G.; Amirul, A.-A.A. Draft genome sequence of the halophilic *Pararhodobacter*-like strain CCB-MM2, which has polyhydroxyalkanoate-synthesizing potential. *Microbiol. Resour. Announc.* **2019**, *8*, doi:10.1128/genomeA.01500–14.
37. Lai, Q.; Liu, X.; Yuan, J.; Xie, S.; Shao, Z. *Pararhodobacter marinus* sp. nov., isolated from deep-sea water of the Indian Ocean. *Int. J. Syst. Evol. Microbiol.* **2019**, *69*, 932–936, doi:10.1099/ijsem.0.003219.
38. Ramana, V.V.; Kumar, P.A.; Srinivas, T.; Sasikala, C.; Ramana, C.V. *Rhodobacter aestuarii* sp. nov., a phototrophic alphaproteobacterium isolated from an estuarine environment. *Int. J. Syst. Evol. Microbiol.* **2009**, *59*, 1133–1136, doi:10.1099/ijs.0.004507-0.
39. Hube, A.E.; Heyduck-Söller, B.; Fischer, U. Phylogenetic classification of heterotrophic bacteria associated with filamentous marine cyanobacteria in culture. *Syst. Appl. Microbiol.* **2009**, *32*, 256–265, doi:10.1016/j.syapm.2009.03.001.
40. Sharifah, E.N.; Eguchi, M. The phytoplankton *Nannochloropsis oculata* enhances the ability of *Roseobacter* clade bacteria to inhibit the growth of fish pathogen *Vibrio anguillarum*. *PloS ONE* **2011**, *6*, doi:10.1371/journal.pone.0026756.
41. van Niel, C.B. The culture, general physiology, morphology, and classification of the non-sulfur purple and brown bacteria. *Bacteriol. Rev.* **1944**, *8*, 1–118.
42. Srinivas, T.; Kumar, P.A.; Sasikala, C.; Ramana, C.V.; Imhoff, J.F. *Rhodobacter vinaykumarii* sp. nov., a marine phototrophic alphaproteobacterium from tidal waters, and emended description of the genus *Rhodobacter*. *Int. J. Syst. Evol. Microbiol.* **2007**, *57*, 1984–1987, doi:10.1099/ijs.0.65077-0.
43. Yu, Y.; Yan, S.-L.; Li, H.-R.; Zhang, X.-H. *Roseicitreum antarcticum* gen. nov., sp. nov., an aerobic bacteriochlorophyll a-containing alphaproteobacterium isolated from Antarctic sandy intertidal sediment. *Int. J. Syst. Evol. Microbiol.* **2011**, *61*, 2173–2179, doi:10.1099/ijs.0.024885-0.
44. Baumann, P.; Baumann, L.; Mandel, M. Taxonomy of marine bacteria: The genus *Beneckea*. *J. Bacteriol.* **1971**, *107*, 268–294.

45. Miyamoto, Y.; Nakamuma, K.; Takizawa, K. Pathogenic halophiles. Proposals of a new genus "*Oceanomonas*" and of the amended species names. *Jpn. J. Microbiol.* **1961**, *5*, 477–481, doi:10.1111/j.1348-0421.1961.tb00225.x.
46. Wang, Y.; Zhang, X.-H.; Yu, M.; Wang, H.; Austin, B. *Vibrio atypicus* sp. nov., isolated from the digestive tract of the Chinese prawn (*Penaeus chinensis* O'sbeck). *Int. J. Syst. Evol. Microbiol.* **2010**, *60*, 2517–2523, doi:10.1099/ijs.0.016915-0.
47. Hugh, R. The proposed conservation of the generic name *Vibrio* pacini 1854 and designation of the neotype strain of *Vibrio cholerae* Pacini 1854. *Int. J. Syst. Evol. Microbiol.* **1964**, *14*, 87–101, doi:10.1099/0096266X-14-2-87.
48. Lee, J.; Shread, P.; Furniss, A.; Bryant, T. Taxonomy and description of *Vibrio fluvialis* sp. nov.(synonym group F vibrios, group EF6). *J. Appl. Microbiol.* **1981**, *50*, 73–94, doi:10.1111/j.1365-2672.1981.tb00873.x.
49. Brenner, D.; Hickman-Brenner, F.; Lee, J.; Steigerwalt, A.; Fanning, G.; Hollis, D.; Farmer, J.; Weaver, R.; Joseph, S.; Seidler, R. *Vibrio furnissii* (formerly aerogenic biogroup of *Vibrio fluvialis*), a new species isolated from human feces and the environment. *J. Clin. Microbiol.* **1983**, *18*, 816–824.
50. Pedersen, K.; Verdonck, L.; Austin, B.; Austin, D.A.; Blanch, A.R.; Grimont, P.A.; Jofre, J.; Koblavi, S.; Larsen, J.L.; Tiainen, T. Taxonomic evidence that V*ibrio carchariae* Grimes et al. 1985 is a junior synonym of *Vibrio harveyi* (Johnson and Shunk 1936) Baumann *et al*. 1981. *Int. J. Syst. Evol. Microbiol.* **1998**, *48*, 749–758, doi:10.1099/00207713-48-3-749.
51. Payne, W.J. Studies on bacterial utilization of uronic acids III.: Induction of oxidative enzymes in a marine isolate. *J. Bacteriol.* **1958**, *76*, 301.
52. Fujino, T. Bacterial food poisoning. *Saishin Igaku* **1951**, *6*, 263–271.
53. Merkel, J.R.; Traganza, E.D.; Mukherjee, B.B.; Griffin, T.B.; Prescott, J. Proteolytic activity and general characteristics of a marine bacterium, *Aeromonas proteolytica* sp. N. *J. Bacteriol.* **1964**, *87*, 1227–1233.
54. Lin, H.; Yu, M.; Wang, X.; Zhang, X.-H. Comparative genomic analysis reveals the evolution and environmental adaptation strategies of vibrios. *BMC Genome* **2018**, *19*, 135, doi:10.1186/s12864-018-4531-2.
55. Tubiash, H.S.; Chanley, P.E.; Leifson, E. Bacillary necrosis, a disease of larval and juvenile bivalve mollusks I. Etiology and epizootiology. *J. Bacteriol.* **1965**, *90*, 1036–1044.
56. Gao, Z.; Ruan, L.; Chen, X.; Zhang, Y.; Xu, X. A novel salt-tolerant endo-β-1, 4-glucanase Cel5A in *Vibrio* sp. G21 isolated from mangrove soil. *Appl. Microbiol. Biotechnol.* **2010**, *87*, 1373–1382, doi:10.1007/s00253-010-2554-y.
57. Ruimy, R.; Breittmayer, V.; Elbaze, P.; Lafay, B.; Boussemart, O.; Gauthier, M.; Christen, R. Phylogenetic analysis and assessment of the genera *Vibrio*, *Photobacterium*, *Aeromonas*, and *Plesiomonas* deduced from small-subunit rRNA sequences. *Int. J. Syst. Evol. Microbiol.* **1994**, *44*, 416–426, doi:10.1099/00207713-44-3-416.
58. Wang, J.-p.; Liu, B.; Liu, G.-h.; Ge, C.-b.; Chen, Q.-q.; Zhu, Y.-j.; Chen, Z. Genome sequence of *Anaerobacillus macyae* JMM-4^T (DSM 16346), the first genomic information of the newly established genus *Anaerobacillus*. *Genome Announc.* **2015**, *3*, doi:10.1128/genomeA.00922–15.
59. Borriss, R.; Chen, X.-H.; Rueckert, C.; Blom, J.; Becker, A.; Baumgarth, B.; Fan, B.; Pukall, R.; Schumann, P.; Spröer, C. Relationship of *Bacillus amyloliquefaciens* clades associated with strains DSM 7^T and FZB42^T: A proposal for *Bacillus amyloliquefaciens* subsp. *amyloliquefaciens* subsp. nov. and *Bacillus amyloliquefaciens* subsp. *plantarum* subsp. nov. based on complete genome sequence comparisons. *Int. J. Syst. Evol. Microbiol.* **2011**, *61*, 1786–1801, doi:10.1099/ijs.0.023267-0.
60. Goto, K.; Omura, T.; Hara, Y.; Sadaie, Y. Application of the partial 16S rDNA sequence as an index for rapid identification of species in the genus *Bacillus*. *J. Gen. Appl. Microbiol.* **2000**, *46*, 1–8, doi:10.2323/jgam.46.1.
61. Ivanova, N.; Sorokin, A.; Anderson, I.; Galleron, N.; Candelon, B.; Kapatral, V.; Bhattacharyya, A.; Reznik, G.; Mikhailova, N.; Lapidus, A. Genome sequence of *Bacillus cereus* and comparative analysis with *Bacillus anthracis*. *Nature* **2003**, *423*, 87–91, doi:10.1038/nature01582.
62. Guinebretière, M.H.; Thompson, F.L.; Sorokin, A.; Normand, P.; Dawyndt, P.; Ehling‐Schulz, M.; Svensson, B.; Sanchis, V.; Nguyen‐The, C.; Heyndrickx, M. Ecological diversification in the *Bacillus cereus* group. *Environ. Microbiol.* **2008**, *10*, 851–865, doi:10.1111/j.1462-2920.2007.01495.x.
63. Massilamany, C.; Mohammed, A.; Loy, J.D.; Purvis, T.; Krishnan, B.; Basavalingappa, R.H.; Kelley, C.M.; Guda, C.; Barletta, R.G.; Moriyama, E.N. Whole genomic sequence analysis of *Bacillus infantis*: Defining the genetic blueprint of strain NRRL B-14911, an emerging cardiopathogenic microbe. *BMC Genome* **2016**, *17*, 511, doi:10.1186/s12864-016-2900-2.
64. Suzuki, T.; Yamasato, K. Phylogeny of spore-forming lactic acid bacteria based on 16S rRNA gene sequences. *FEMS Microbiol. Lett.* **1994**, *115*, 13–17, doi:10.1111/j.1574-6968.1994.tb06607.x.
65. Nielsen, P.; Rainey, F.A.; Outtrup, H.; Priest, F.G.; Fritze, D. Comparative 16S rDNA sequence analysis of some alkaliphilic bacilli and the establishment of a sixth rRNA group within the genus *Bacillus*. *FEMS Microbiol. Lett.* **1994**, *117*, 61–65, doi:10.1111/j.1574-6968.1994.tb06736.x.

66. Chelliah, R.; Wei, S.; Park, B.-J.; Rubab, M.; Dalirii, E.B.-M.; Barathikannan, K.; Jin, Y.-G.; Oh, D.-H. Whole genome sequence of *Bacillus thuringiensis* ATCC 10792 and improved discrimination of *Bacillus thuringiensis* from *Bacillus cereus* group based on novel biomarkers. *Microb. Pathog.* **2019**, *129*, 284–297, doi:10.1016/j.micpath.2019.02.014.
67. Zhao, J.-Y.; Geng, S.; Xu, L.; Hu, B.; Sun, J.-Q.; Nie, Y.; Tang, Y.-Q.; Wu, X.-L. Complete genome sequence of *Defluviimonas alba* cai42^T, a microbial exopolysaccharides producer. *J. Biotechnol.* **2016**, *239*, 9–12, doi:10.1016/j.jbiotec.2016.09.017.
68. Anton, B.P.; Roberts, R.J.; Fomenkov, A.; Humbert, A.; Stoian, N.; Zeilstra-Ryalls, J. Complete genome sequences of two *Rhodobacter* strains. *Microbiol. Resour. Announc.* **2018**, *7*, doi:10.1128/MRA.01162–18.
69. Wang, P.; Wen, Z.; Li, B.; Zeng, Z.; Wang, X. Complete genome sequence of *Vibrio alginolyticus* ATCC 33787^T isolated from seawater with three native megaplasmids. *Mar. Genome* **2016**, *28*, 45–47, doi:10.1016/j.margen.2016.05.003.
70. Liu, X.-F.; Cao, Y.; Zhang, H.-L.; Chen, Y.-J.; Hu, C.-J. Complete genome sequence of *Vibrio alginolyticus* ATCC 17749^T. *Genome Announc.* **2015**, *3*, doi:10.1128/genomeA.01500–14.
71. Aznar, R.; Ludwig, W.; Amann, R.; Schleifer, K. Sequence determination of rRNA genes of pathogenic *Vibrio* species and whole-cell identification of *Vibrio vulnificus* with rRNA-targeted oligonucleotide probes. *Int. J. Syst. Evol. Microbiol.* **1994**, *44*, 330–337, doi:10.1099/00207713-44-2-330.
72. Sichtig, H.; Minogue, T.; Yan, Y.; Stefan, C.; Hall, A.; Tallon, L.; Sadzewicz, L.; Nadendla, S.; Klimke, W.; Hatcher, E. FDA-ARGOS is a database with public quality-controlled reference genomes for diagnostic use and regulatory science. *Nat. Commun.* **2019**, *10*, 1–13, doi:10.1038/s41467-019-11306-6.
73. Weinstock, M.T.; Hesek, E.D.; Wilson, C.M.; Gibson, D.G. *Vibrio natriegens* as a fast-growing host for molecular biology. *Nat. Methods* **2016**, *13*, 849–851, doi:10.1038/nmeth.3970.
74. Richards, G.P.; Needleman, D.S.; Watson, M.A.; Bono, J.L. Complete genome sequence of the larval shellfish pathogen *Vibrio tubiashii* type strain ATCC 19109. *Genome Announc.* **2014**, *2*, doi:10.1128/genomeA.01252-14.
75. Hernández-González, I.L.; Olmedo-Álvarez, G. Draft whole-genome sequence of the type strain *Bacillus aquimaris* TF12^T. *Genome Announc.* **2016**, *4*, doi:10.1128/genomeA.00640-16.
76. Dunlap, C.A.; Saunders, L.P.; Schisler, D.A.; Leathers, T.D.; Naeem, N.; Cohan, F.M.; Rooney, A.P. *Bacillus nakamurai* sp. nov., a black-pigment-producing strain. *Int. J. Syst. Evol. Microbiol.* **2016**, *66*, 2987–2991, doi:10.1099/ijsem.0.001135.
77. Lapidus, A.; Goltsman, E.; Auger, S.; Galleron, N.; Ségurens, B.; Dossat, C.; Land, M.L.; Broussolle, V.; Brillard, J.; Guinebretiere, M.-H. Extending the *Bacillus cereus* group genomics to putative food-borne pathogens of different toxicity. *Chem. Biol. Interact.* **2008**, *171*, 236–249, doi:10.1016/j.cbi.2007.03.003.
78. Wang, J.-p.; Liu, B.; Liu, G.-h.; Chen, D.-j.; Chen, Q.-q.; Zhu, Y.-j.; Chen, Z.; Che, J.-m. Draft genome sequence of *Bacillus marisflavi* TF-11^T (JCM 11544), a carotenoid-producing bacterium isolated from seawater from a tidal flat in the Yellow Sea. *Genome Announc.* **2015**, *3*, doi:10.1128/genomeA.01451-15.
79. Johnson, S.L.; Daligault, H.E.; Davenport, K.W.; Jaissle, J.; Frey, K.G.; Ladner, J.T.; Broomall, S.M.; Bishop-Lilly, K.A.; Bruce, D.C.; Gibbons, H.S. Complete genome sequences for 35 biothreat assay-relevant *Bacillus* species. *Genome Announc.* **2015**, *3*, doi:10.1128/genomeA.00151-15.
80. Whitman, W.B.; Woyke, T.; Klenk, H.-P.; Zhou, Y.; Lilburn, T.G.; Beck, B.J.; De Vos, P.; Vandamme, P.; Eisen, J.A.; Garrity, G. Genomic encyclopedia of bacterial and archaeal type strains, phase III: The genomes of soil and plant-associated and newly described type strains. *Stand. Genome Sci.* **2015**, *10*, 1–6, doi:10.1186/s40793-015-0017-x.
81. Wang, J.-p.; Liu, B.; Liu, G.-h.; Ge, C.-b.; Xiao, R.-f.; Zheng, X.-f.; Shi, H. Draft genome sequence of *Bacillus shackletonii* LMG 18435^T, isolated from volcanic mossy soil. *Genome Announc.* **2016**, *4*, doi:10.1128/genomeA.01689-15.
82. Nye, T.M.; Schroeder, J.W.; Kearns, D.B.; Simmons, L.A. Complete genome sequence of undomesticated *Bacillus subtilis* strain NCIB 3610. *Genome Announc.* **2017**, *5*, doi:10.1128/genomeA.00364-17.
83. Kawato, S.; Nozaki, R.; Kondo, H.; Hirono, I. Draft genome sequences of *Vibrio atypicus* strains DSM 25292^T and TUMSAT1. *Microbiol. Resour. Announc.* **2020**, *9*, doi:10.1128/MRA.01526-19.
84. Knoll, M.; Hamm, T.M.; Wagner, F.; Martinez, V.; Pleiss, J. The PHA Depolymerase Engineering Database: A systematic analysis tool for the diverse family of polyhydroxyalkanoate (PHA) depolymerases. *BMC Bioinform.* **2009**, *10*, 89, doi:10.1186/1471-2105-10-89.
85. Van Pouderoyen, G.; Eggert, T.; Jaeger, K.-E.; Dijkstra, B.W. The crystal structure of *Bacillus subtilis* lipase: A minimal α/β hydrolase fold enzyme. *J. Mol. Biol.* **2001**, *309*, 215–226, doi:10.1006/jmbi.2001.4659.
86. Papageorgiou, A.C.; Hermawan, S.; Singh, C.B.; Jendrossek, D. Structural basis of poly (3-hydroxybutyrate) hydrolysis by PhaZ7 depolymerase from *Paucimonas lemoignei*. *J. Mol. Biol.* **2008**, *382*, 1184–1194, doi:10.1016/j.jmb.2008.07.078.

87. Wakadkar, S.; Hermawan, S.; Jendrossek, D.; Papageorgiou, A.C. The structure of PhaZ7 at atomic (1.2 Å) resolution reveals details of the active site and suggests a substrate-binding mode. *Acta Cryst.* **2010**, *F66*, 648–654, doi:10.1107/S174430911001434X.
88. Jendrossek, D.; Hermawan, S.; Subedi, B.; Papageorgiou, A.C. Biochemical analysis and structure determination of *Paucimonas lemoignei* poly (3‐hydroxybutyrate) (PHB) depolymerase PhaZ 7 muteins reveal the PHB binding site and details of substrate–enzyme interactions. *Mol. Biotechnol.* **2013**, *90*, 649–664, doi:10.1111/mmi.12391.
89. Leow, T.C.; Rahman, R.N.Z.R.A.; Basri, M.; Salleh, A.B. High level expression of thermostable lipase from *Geobacillus* sp. strain T1. *Biosci. Biotech. Bioch.* **2004**, *68*, 96–103, doi:10.1271/bbb.68.96.
90. Matsumura, H.; Yamamoto, T.; Leow, T.C.; Mori, T.; Salleh, A.B.; Basri, M.; Inoue, T.; Kai, Y.; Rahman, R.N.Z.R.A. Novel cation‐π interaction revealed by crystal structure of thermoalkalophilic lipase. *Proteins* **2008**, *70*, 592–598, doi:10.1002/prot.21799.
91. Sharma, P.K.; Mohanan, N.; Sidhu, R.; Levin, D.B. Colonization and degradation of polyhydroxyalkanoates by lipase-producing bacteria. *Can. J. Microbiol.* **2019**, *65*, 461–475, doi:10.1139/cjm-2019-0042.
92. Jørgensen, S.; Skov, K.; Diderichsen, B. Cloning, sequence, and expression of a lipase gene from *Pseudomonas cepacia*: Lipase production in heterologous hosts requires two *Pseudomonas* genes. *J. Bacteriol.* **1991**, *173*, 559–567, doi:10.1128/jb.173.2.559-567.1991.
93. Schrag, J.D.; Li, Y.; Cygler, M.; Lang, D.; Burgdorf, T.; Hecht, H.-J.; Schmid, R.; Schomburg, D.; Rydel, T.J.; Oliver, J.D. The open conformation of a *Pseudomonas* lipase. *Structure* **1997**, *5*, 187–202, doi:10.1016/S0969-2126(97)00178-0.
94. Taipa, M.A.; Liebeton, K.; Costa, J.V.; Cabral, J.M.; Jaeger, K.-E. Lipase from *Chromobacterium viscosum*: Biochemical characterization indicating homology to the lipase from *Pseudomonas glumae*. *Biochim. Biophys. Acta (BBA)-Lipids Lipid Metab.* **1995**, *1256*, 396–402, doi:10.1016/0005-2760(95)00052-E.
95. Lang, D.; Hofmann, B.; Haalck, L.; Hecht, H.-J.; Spener, F.; Schmid, R.D.; Schomburg, D. Crystal structure of a bacterial lipase from *Chromobacterium viscosum* ATCC 6918 refined at 1.6 A resolution. *J. Mol. Biol.* **1996**, *259*, 704–717, doi:10.1006/jmbi.1996.0352.
96. Jaeger, K.-E.; Adrian, F.-J.; Meyer, H.E.; Hancock, R.E.; Winkler, U.K. Extracellular lipase from *Pseudomonas aeruginosa* is an amphiphilic protein. *Biochim. Et Biophys. Acta (BBA)-Protein Struct. Mol. Enzymol.* **1992**, *1120*, 315–321, doi:10.1016/0167-4838(92)90254-B.
97. Nardini, M.; Lang, D.A.; Liebeton, K.; Jaeger, K.-E.; Dijkstra, B.W. Crystal structure of *Pseudomonas aeruginosa* lipase in the open conformation. The prototype for family I. 1 of bacterial lipases. *J. Biol. Chem.* **2000**, *275*, 31219–31225, doi:10.1074/jbc.M003903200.

Publisher's Note: MDPI stays neutral with regard to jurisdictional claims in published maps and institutional affiliations.

© 2020 by the authors. Submitted for possible open access publication under the terms and conditions of the Creative Commons Attribution (CC BY) license (http://creativecommons.org/licenses/by/4.0/).
